# Hapten Mediated Display and Pairing of Recombinant Antibodies Accelerates Assay Assembly for Biothreat Countermeasures

**DOI:** 10.1038/srep00807

**Published:** 2012-11-12

**Authors:** Laura J. Sherwood, Andrew Hayhurst

**Affiliations:** 1 Department of Virology and Immunology, Texas Biomedical Research Institute, San Antonio, Texas, USA

## Abstract

A bottle-neck in recombinant antibody sandwich immunoassay development is pairing, demanding protein purification and modification to distinguish captor from tracer. We developed a simple pairing scheme using microliter amounts of *E. coli* osmotic shockates bearing site-specific biotinylated antibodies and demonstrated proof of principle with a single domain antibody (sdAb) that is both captor and tracer for polyvalent *Marburgvirus* nucleoprotein. The system could also host pairs of different sdAb specific for the 7 botulinum neurotoxin (BoNT) serotypes, enabling recognition of the cognate serotype. Inducible *supE* co-expression enabled sdAb populations to be propagated as either phage for more panning from repertoires or expressed as soluble sdAb for screening within a single host strain. When combined with streptavidin-g3p fusions, a novel transdisplay system was formulated to retrofit a semi-synthetic sdAb library which was mined for an anti-*Ebolavirus* sdAb which was immediately immunoassay ready, thereby speeding up the recombinant antibody discovery and utilization processes.

Rapidly generating diagnostic and environmental surveillance tests to emerging biothreats is one route to supplementing the toolbox of countermeasures required to help safeguard human health. Making recombinant antibodies to formulate such immunoassays has many advantages over classical hybridoma technology or polyclonal sera generation including the ability to select for the desired levels of specificity/cross-reactivity and affinity, and employ directed evolution to enhance these properties further (for review see^1^). Currently, several methods exist for selecting recombinant antibodies from repertoires including phage, yeast and ribosome display among the more popular. The essence of each method is a link of each antibody phenotype to its genotype to allow antigen binding clones to be enriched from a large starting panel of antibodies. The panels can be generated by cloning the variable domains from hosts immunized with the antigen of interest; usually several million clones are sufficient to ensure antigen reactive clones are represented. Alternatively the panel can be made by cloning variable domains from a sufficiently high number of non-immune hosts, or assembling synthetic or semi-synthetic repertoires with artificially diversified antigen contact loops; usually several billion (1e+9 and upwards) is required to ensure antigen binding clones with reasonably high affinities can be reliably isolated. The latter approach enables the same panel of antibodies (a “single pot” library) to be used for multiple antigens of interest and since inception^2^ has become both ethically and financially appealing as well as fast since it bypasses several weeks to months required to generate an immune response and clone the resulting repertoire. Phage display is typically used for these larger libraries since it is straightforward to use and multiple representations of each clone can be present in small volumes owing to the size of the phage particles, thereby enabling multiple parallel selections with minimal equipment. Selections typically involve allowing the phage panels to bind immobilized antigen, eluting the binders and amplifying them in *E. coli* and repeating the process until a sufficient percentage of the population are antigen-specific and give rise to a polyclonal phage ELISA positive signal. Single clones are then usually identified by picking individual members of the polyclonal population and performing monoclonal phage ELISA in 96 well plates and then sequencing the positives to identify unique clones.

However, whether one uses an immune approach or a single-pot phage display approach, there is a need for streamlined ways to characterize the resultant unique antibody proteins in a format that closely resembles their intended use. For diagnostic and environmental detection purposes this is typically a form of antigen capture assay where one antibody is the captor and one antibody is the tracer. An example of this type of characterization was part of our work developing highly specific capture assays to each of the seven botulinum neurotoxin (BoNT) serotypes from a llama that had been immunized with non-toxic toxoid versions of the neurotoxins^3^. The resulting multiplex immunization generated over 130 different single domain antibody (sdAb) clones, each requiring expression in shake flask cultures to generate sufficient material for immobilized metal affinity chromatography followed by gel filtration. Each purified protein was then covalently attached to microbeads to form the captor and each was also chemically biotinylated to form the tracer, enabling each antibody to be checker-boarded with each other to identify pairs of non-competitive clones in a liquid microarray. Each purification in sets of four takes the best part of two days, subsequent modifications can be done in larger batches and take another day or so, yet the whole process is labor intensive, prone to mix-ups, costly and time-consuming taking over a year in total. Likewise to form a typical antigen capture ELISA the purified captor antibody would need to be passively or chemically immobilized to a surface, while the tracer would still need to be made chemically distinct to enable the reporter fluorophore or enzyme conjugate to distinguish it from the captor. The tracer can also be fused genetically or chemically to a reporter enzyme or fluorescent protein and though straightforward, these can again be cost and time burdens taking several days to generate and purify, and are usually only applied to the final chosen antibody clones rather than entire panels. While higher throughput methods of antibody purification exist to handle 96 cultures of a few mL^4^ to several hundred mL culture volumes^5^ to a litre (http://www.lanl.gov/orgs/b/pdfs/LAMRA.pdf), these are not always affordable by the average academic laboratory. Monitoring the direct binding of these pure unmodified recombinant antibodies to antigen *via* a range of biosensors could be used in competition screens (“epitope binning”^6^), though once again, these instruments are very costly and the assay is also based on the assumption that immobilized antigen faithfully represents that in solution, which may not necessarily be the case. Ideally, characterizing panels of recombinant antibodies from phage display libraries intended for sandwich assay formulation would benefit from a system that bypasses the need for protein purification as well as modification, yet enables the clones to be simply screened in pairs for binding solution phase antigen.

Such systems have been formulated to screen pairs of mouse monoclonal antibodies (Mab) from hybridoma supernatants and rely upon either protein A or an anti-Fc antibody to act as a generic high affinity sink for captor and tracer antibodies^7^,^8^,^9^. Although IgG antibodies to peptide tags typically appended to recombinant antibody genes in phage display vectors like the myc tag^10^ and the hexahistidine tag^11^ might be capable of enabling such a system for our needs, these are costly, introduce a relatively fragile IgG protein into the system and also may not have sufficient affinity to prevent drift during the course of the assay. For these reasons we explored the potential of the million fold higher affinity biotin/avidin interaction to act as a foundation for allowing us to rapidly pair recombinant antibodies destined for sandwich assays.

Although site specific biotinylation of recombinant antibodies has been used for many years^12^,^13^, it is often thought that when devising a sandwich assay based upon them that the tracer must also be made additionally distinct from the captor either by fusion to a different epitope tag or a reporter enzyme (e.g.^14^,^15^) following the conventions described above. We hypothesized that if the single biotin associated with the captor recombinant antibody is correctly bound by a neutravidin coated platform, then the same style of singly biotinylated antibody can be used as tracer since the subsequent neutravidin enzyme secondary conjugate will only recognize the free tracer biotin and not the occluded biotin on the captor. It is important to note that this will only be the case when a single biotin is incorporated into the antibody and is unlikely to work on chemically biotinylated material where multiple biotins are randomly distributed over the surface of the protein. The high affinities that both avidin and streptavidin have for biotin and the almost quantitative concealment of the bound hapten^16^ should ensure an essentially irreversible captor platform with no background binding. Being very thermostable proteins^17^, avidin and streptavidin also fulfill our long term goals of incorporating the clones into rugged assays that bypass the need for cold-chains thereby enabling storage in resource limited settings devoid of regular electricity supply. We sought to explore this idea in the context of phage display systems with the goal of delivering stop-gap antigen capture assays to emerging biological threats as fast as possible. A crucial feature of the system was independence from DNA sequencing or antibody purification to speed up the assay pipeline in high containment environments, thereby focusing downstream resources on the most appropriate clones. In terms of speed and ease of process, from the time the expression culture is harvested to the results are generated can take as little as 4 h with nothing more complicated than an ELISA plate for equipment.

Figure 1 shows the principle of the antibody pairing system using polyvalent *Marburgvirus* nucleoprotein as a model antigen and a single sdAb (MBG B) that was previously shown to perform as both captor and tracer^18^. Such a set-up is the acid test for pairing since a single crude osmotic shockate sample of *E. coli* expressing the sdAb is used as the sole source for both antigen capture and antigen tracing, with distinction by the secondary enzyme conjugate defined on the ratio of signal to noise. The essence of the method is the stepwise occlusion of the biotin on the captor antibody before the tracer antibody is utilized.

Though the rapid antibody pairing system was the main focus of this work, we also aimed to streamline the route for antibody protein expression itself to simplify the process further. Typically, most phage display vectors employ a genetic switch between the antibody gene and phage coat protein gene in the form of an amber codon^19^. Phage display is performed in host *E. coli* cells that contain a constitutively expressed suppressor tRNA that enables a small portion of the amber codons to be translated to enable read-through of the sdAb to the desired phage coat protein gene, thereby generating the fusion protein required for assembly into the phage particles. Following selection of clones as phage displayed motifs the genes are mobilized to a second host devoid of the suppressor tRNA to enable high level expression of just the antibody gene, since the whole fusion is often cytocidal and prevents optimal expression conditions from being attained^20^. To bypass the need for this transfer we engineered a novel *E. coli* host strain that is able to inducibly express the suppressor tRNA thereby enabling phage display to be performed when “on” or high level antibody production when “off”. The new strain eliminates human error when mobilizing large numbers of clones from suppressor positive to negative hosts, accelerates the characterization process, halves archival storage space and eliminates immunity/clonal toxicity concerns when using strong promoters^21^.

We further developed this theme of streamlining the antibody generation process with a view to developing a novel phage based display system where the machinery for both inducible suppression and phage display is on one vector while the biotinylated recombinant antibody expression cassette is on another. Our goal here was to minimize the size of the vector that is used to assemble the antibody gene repertoires to improve transformation efficiencies and enable larger libraries to be made, a very important caveat for single-pot systems. Here, the necessary link between antibody phenotype and genotype is *via* the single biotin hapten which is bound in *trans* by a recombinant streptavidin expressed as the fusion to the minor phage coat protein. While many examples of protein-protein interactions exist to enable phage display, including covalent cross-linking of cysteine^22^ or using peptide zippers^23^ our system of transdisplay mediated *via* a hapten, has to our knowledge, not been described previously.

Herein we have developed these three new systems to act in concert and applied them to biological threat agents of particular interest to us, though there is no *a priori* reason why they should not be capable of being applied to other antigens. For us, the botulinum neurotoxins (BoNT) regarded as “the most poisonous poison”^24^ represent model biosafety level two antigens lethal at µg-pg/kg body weight depending on the serotype, route of administration and whether the material is present in toxin or complex form (for review see^25^). Our previous immune library and resulting heptaplex panel of sdAb specific to all seven serotypes of toxin and toxin complexes^3^ enabled us to path-find several aspects of the new systems. Our other main target group comprises the Filoviruses, *Marburgvirus* and *Ebolavirus* which, depending on the particular species, have the ability to cause diseases which are often fatal and can manifest themselves as severe hemorrhagic fevers (for review see^26^). With no licensed vaccines or therapeutics available these agents are restricted to the biosafety level 4 laboratory, and *via* our existing semi-synthetic single-pot derived anti-Filoviral sdAb^18^,^27^ provide us with model antigens to path-find the applicability of the antibody selection and pairing scheme to high containment environments. While Filoviruses and botulinum neurotoxins are fascinating in their own rights as exotic viral and protein based nanomachines, they are also on the CDC category A list of biological threat agents as potential mass casualty weapons and so fit in well with our broad long-term objective of developing disruptive countermeasures to high consequence pathogens and toxins.

## Results

### Establishing constructs and conditions for proof of principle rapid antibody pairing

**Figure 2:**
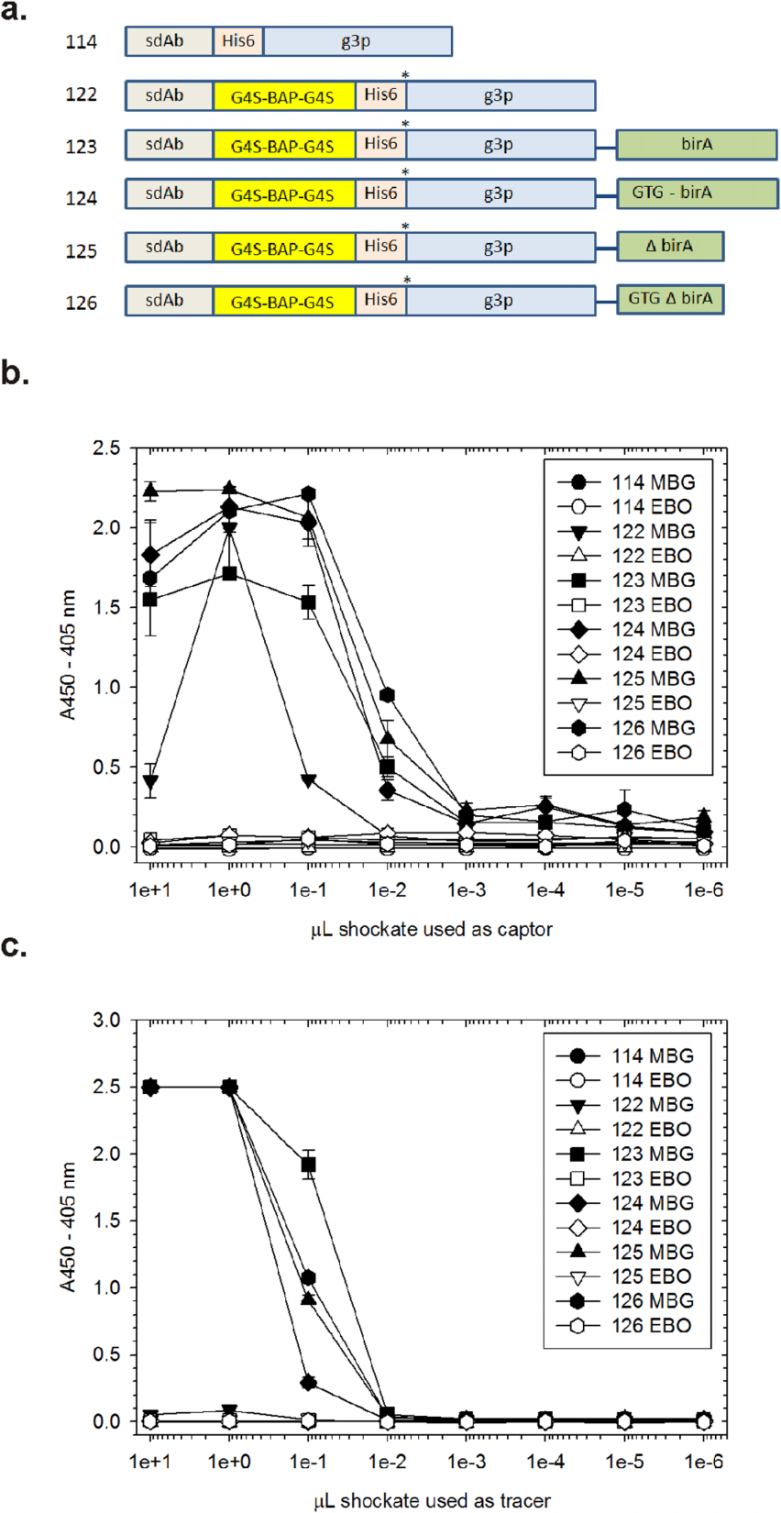
Development of rapid antibody pairing vectors compatible with phage display and demonstrating proof of principle assays. (a) Vector designs used to determine the requirements for the biotin acceptor peptide (BAP) and downstream biotin ligase (birA) cistron^60^ for pairing. Each cassette is borne on a high copy number ampicillin resistant phagemid backbone based upon a tac promoter version of pecan45^3^ and the sdAb cistron is signaled to the periplasm *via* a pelB leader sequence (details of construction can be found in the methods section online). Pecan114 is the parental sdAb phagemid display vector with an amber codon (*) between the sdAb-His6 gene and M13K07 gene III (g3p). Pecan 122–126 have the minimal biotin acceptor peptide (BAP)^61^ inserted between the sdAb and His6 tag, flanked at either end with a sequence encoding gly4ser (G4S). A full-length birA gene or a DNA binding domain deletion (ΔbirA)^62^ was inserted downstream of g3p in pecan123 and pecan 125 respectively and its initiation codon was altered to the less powerful GTG in pecan 124 and 126. (b) Demonstrating sdAb capture: varying amounts of osmotic shockates from IPTG induced cultures of the commonly used non-suppressor host HB2151 bearing each of the vectors were used as captor with fixed amounts of XL1-Blue generated phage supernatants. MBG and EBO signify the antigens used were either *Marburgvirus* or *Ebolavirus* Zaire polyvalent nucleoprotein preparations^63^ respectively. (Supplementary Figure S1 demonstrates the antigens used are specifically capable of polyvalent binding with conventionally immobilized non-biotinylated sdAb and sdAb-alkaline phosphatase tracers^18^). (c) Demonstrating sdAb capture and tracing ie. pairing : employing a fixed amount (1 µL) of shockate as captor and varying amounts of shockate as tracer reveals pairing of sdAb is possible from unpurified samples to enable specific recognition of target antigen.

Figure 2a shows the initial construct designs used to empirically balance *in vivo* sdAb biotinylation with phage display compatibility and effectiveness of capturing/tracing. The vectors were first employed in *E. coli* strain HB2151 to generate small-scale periplasmic extracts *via* osmotic shocking to examine if sufficient biotinylated antibody could be generated for capture by neutravidin. Osmotic shocking is a very simple means of forcing the contents of the periplasm out of the cell by weakening the peptidoglycan with a Tris-EDTA-lysozyme mix while the cells are under a mild hyperosmotic shock of sucrose. Since the bulk of host proteins are in the cytosol it is an ideal way of preferentially enriching for recombinant antibody fragments exported to the periplasm, as is the case here. Titrating the osmotic shockates over a fixed amount of neutravidin, incubating with antigen and then using a constant amount of phage displayed sdAb as tracers (10 µL of supernatant from M13K07 infected XL1-Blue cultures) demonstrates the requirement for the biotin acceptor peptide (BAP) sequence for sdAb capture (compare vectors pecan114 and 126 in Fig. 2b). Superior signals were obtained when a biotin ligase (*birA*) cistron was included in addition to the BAP (pecan123-126) especially when the birA DNA binding domain was absent and a poor translation initiation signal was employed (pecan126).

Having shown the captor portion of the assay was working, we then proceeded to replace the phage tracer with various amounts of the osmotic shockates, thus using the biotinylated sdAb as *both* captor and tracer. Using a constant 1 µL of the shockate as captor, and titrating the shockate as tracer reveals the rapid antibody pairing system works well though with a 10-100 fold drop in sensitivity from phagemid tracer (Fig. 2c). The phagemid body is comprised of over a thousand copies of the major coat protein and affords an easy way of amplifying ELISA signals when using anti-M13 major coat protein HRP conjugates for detection^2^. The data also shows the absolute requirement for the 2^nd^ cistron birA in the face of this signal decrease when only a single neutravidin-HRP is expected to bind each tracer sdAb. Note the complete absence of background ELISA signal when using negative control antigen *Ebolavirus* Zaire nucleoprotein, indicating the pairing system is very clean, with secondary conjugate not appearing to detect rogue immobilized captor sdAb or background binding tracer sdAb. Though full-length ATG initiated birA (pecan123) may have a mild advantage when using sdAb vs sdAb pairing, the drop in signal when using it for sdAb vs phage pairing indicated potential incompatibility with effective phage display (confirmed in Supplementary Fig. S2), thus only the GTGΔbirA platform was pursued further.

### Engineering a single host strain that facilitates both rapid antibody pairing and phage display

**Figure 3:**
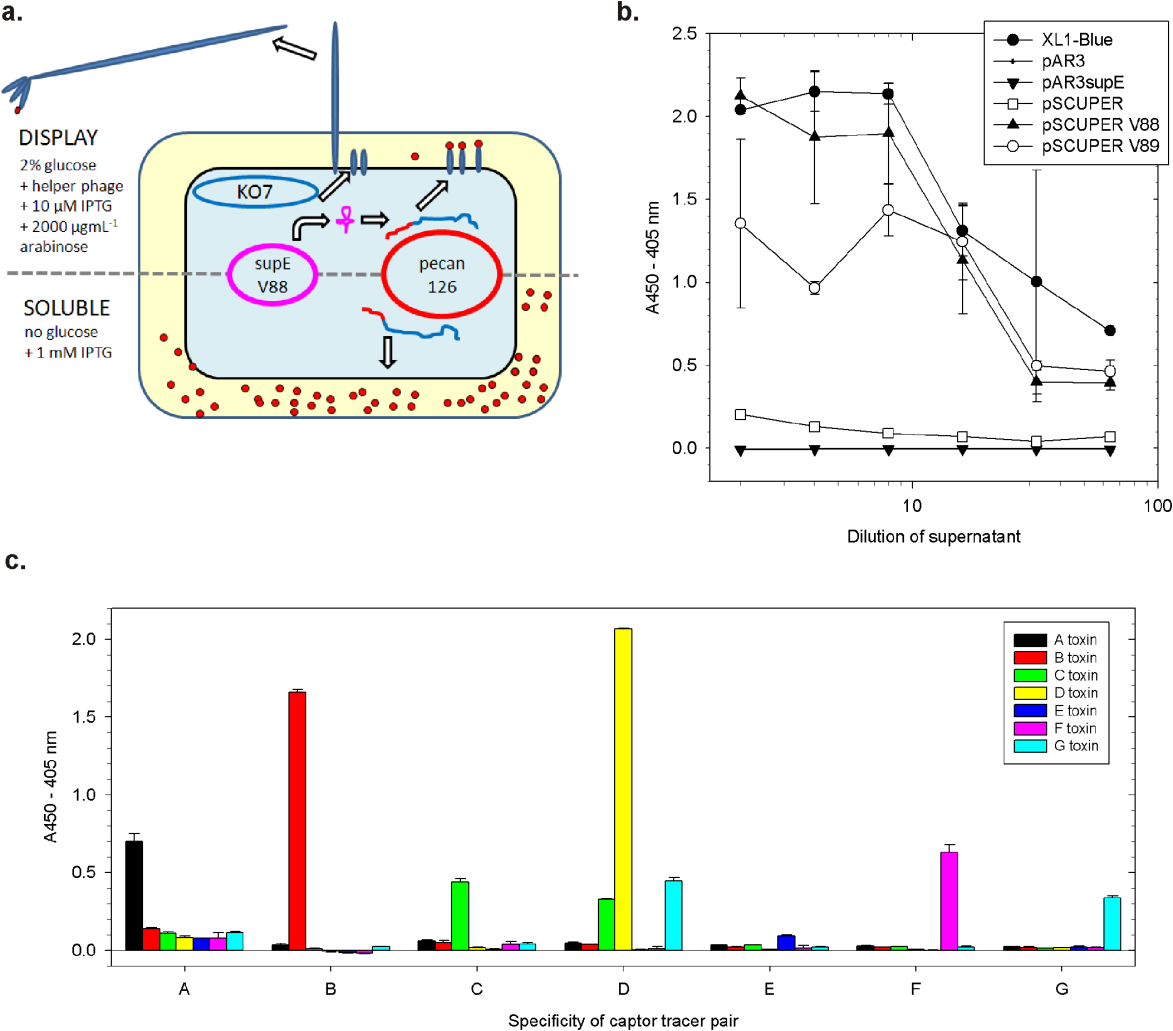
Conversion of non-suppressor host strain HB2151 to a conditional suppressor strain HBV88 that enables both rapid antibody pairing and phage display. (a) Schematic showing host HBV88 in phage display mode (above dashed line) where arabinose induction of plasmid borne *supE* in the presence of glucose^64^ enables supE-V88 expression and subsequent suppression of sdAb-g3p fusion protein translation. In soluble expression mode (below dashed line), the amber codon is not suppressed and full induction with IPTG in the absence of glucose generates large amounts of soluble periplasmic sdAb. (b) Engineering a suitable arabinose inducible *supE* vehicle to enable HBV88 to work is assayed by phage ELISA of pecan114 display on His-tagged *Marburgvirus* nucleoprotein. Negative control signals were ranged from 0.000 to −0.007 on an equivalent amount of His-tagged *Ebolavirus* Zaire nucleoprotein. Equivalent rankings were also obtained in DH10BF' (Supplementary Fig. S3) showing that we can conditionally transform other non-suppressor hosts into *supE* positive display hosts. (c) Pairing of the 14 anti-BoNT specific sdAb combinations from 10 µL of osmotic shockates from HBV88 in soluble sdAb expression mode bearing pecan132 vectors and detecting a standard 1 µgmL^−1^ concentration of each serotype demonstrates that each pair essentially retains specificity with minor cross-reactivity between D and C owing to the mosaic nature of the D used.

A schematic of the strain is shown in Figure 3a and relies upon the provision of an inducible synthetic suppressor tRNA^28^ that conditionally reads through the amber codon of commonly used display vectors^19^. We first chose pAR3^29^ as a vehicle for *supE*, which is a low copy number p15a origin chloramphenicol resistant arabinose inducible plasmid that is compatible with the colE1 origin of the display phagemids. Though M13K07 helper phage also has a p15a origin, the different resistance genes between phage and plasmid should allow both to be selected for within the same cell, a feature we have used previously for same origin vehicles^30^. However, when compared with *E. coli* XL-1 Blue, a commonly used phage display host that is constitutively *supE* positive, we could not detect suppression mediated by pAR3-supE within HB2151 bearing the basal display vector pecan114 (Fig. 3b). Therefore, we elevated the supressor gene dosage by fusing the chloramphenicol resistance/arabinose inducible *supE* region of pAR3-supE with the origin of an elevated copy number variant of pSC101^31^ to create pSCUPER which enabled low but detectable display. To increase display further we separately introduced the V88 and V89 mutations previously shown to improve *supE* mediated suppression in streptomycin resistant hosts^32^ resulting in levels of display equivalent to XL1-Blue at least for V88. The resulting HB2151 based strain of HB2151+pSCUPER-V88 was denoted HBV88 and was first employed to study variants of the linkers flanking the BAP sequence for impact on display to reveal both linkers appear advantageous (Supplementary Fig. S2). The absence of the trailing Gly4Ser caused an approximate 3 fold drop in soluble sdAb expression (Supplementary Fig. S4), though all constructs generated highly pure sdAb as judged by Coomassie stained SDS-PAGE (Supplementary Fig. S5) with mass-spectrometry of the peak fractions revealing unbiotinylated and biotinylated sAb (Supplementary Fig. S6). All of the rapid antibody pairing constructs when mobilized to HBV88 enabled sdAb pairing (Supplementary Fig. S7) thus confirming the dual utility of the new host strain. One vector pecan132, was used within HBV88 to host a panel of sdAb that form specific pairs capable of recognizing each of the 7 BoNT toxins^3^ to confirm recognition of the cognate toxin serotype using just crude shockates (Fig. 3c). That serotype E is yielding a low signal may in part be due to the fact that this pair was our lowest sensitivity combination within the panel of 7 sdAb pairs though it may also reflect the fact that the initial screening by covalent modification did not necessarily identify the best performing clones by rapid antibody pairing. Noteworthy is the conservation of low cross-reactivity shown by the D mosaic pair for serotype G, reminding us that serotyping of BoNT proteins is complex with several examples of cross-reactive monoclonal antibodies^33^,^34^, polyclonal animal and human sera^35^,^36^ (and references in^3^ and^37^).

### Engineering a novel phage transdisplay system that capitalises on rapid antibody pairing mechanics

**Figure 4:**
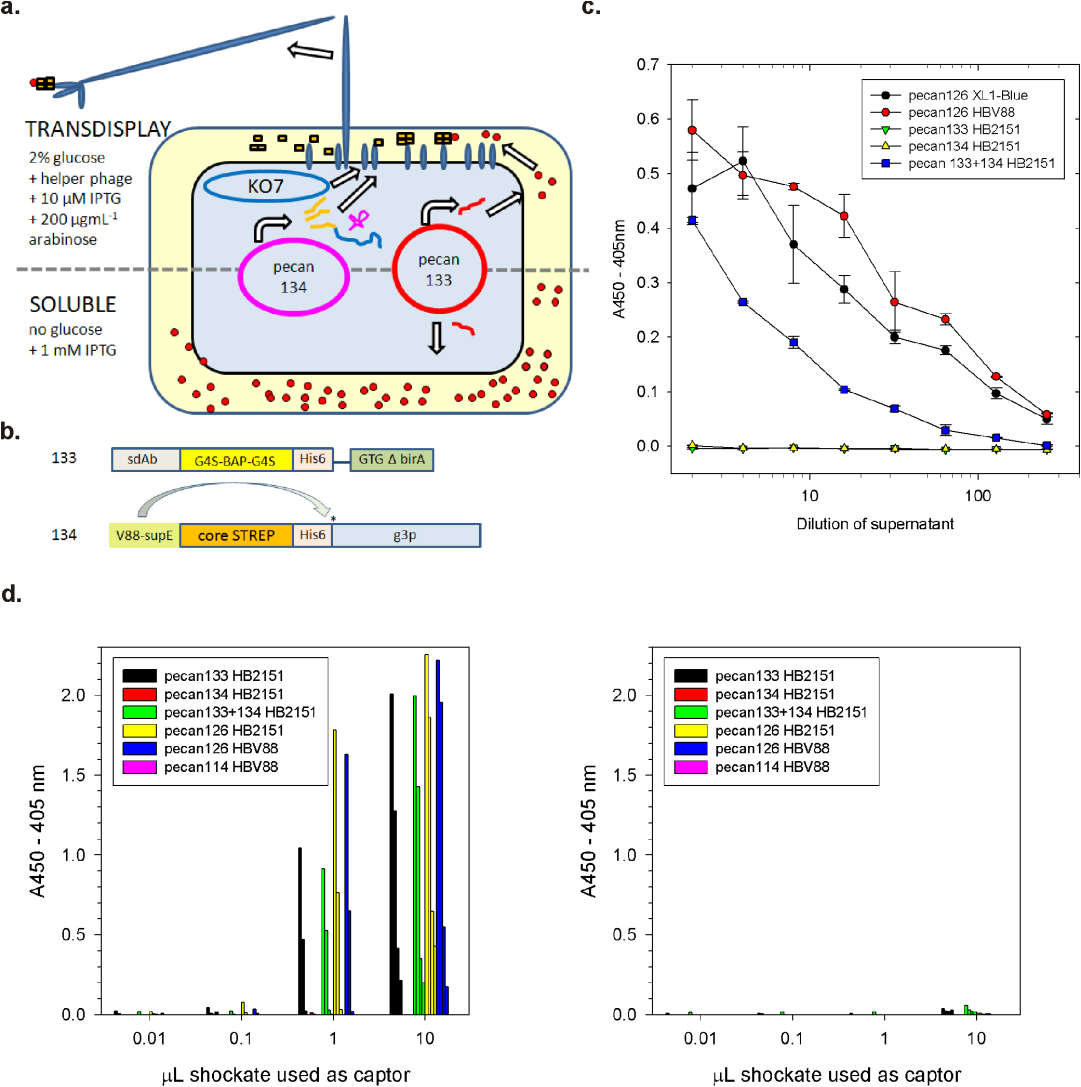
Development of a novel plasmid plus phagemid transdisplay system that employs streptavidin-g3p to indirectly display *in vivo* biotinylated periplasmic sdAb. (a) Schematic of the transdisplay system where pecan134, a chloramphenicol resistant pSC101 high copy number origin mutant plasmid expresses supEV88 which suppresses a streptavidin core minigene fused to g3p platform, while pecan133 is an ampicillin resistant phagemid that expresses sdAb-BAP and birA only. Induction for soluble sdAb expression is the same as the HBV88 system, though for display the arabinose concentration is 10 fold lower to reduce toxicity from strep-g3p over expression. (b) Schematic of the basic cassettes used for transdisplay with pecan133 driven by the IPTG inducible tac promoter and pecan134 by an arabinose promoter with the amber codon (*). (c) Monoclonal phage ELISA on *Marburgvirus* NP to compare conventional display of MBG B sdAb (pecan126 XL1-Blue), HBV88 mediated display (pecan126 HBV88), separate transdisplay components (pecan133 HB2151 and pecan134 HB2151) and combined transdisplay components (pecan133+134 HB2151). Signals on negative control *Ebolavirus* NP antigen ranged from 0.017 to −0.007. (d) Pairing of small-scale shockates from constructs and strains in soluble sdAb expression to demonstrate that the transdisplay vector pecan133 is equivalent to the conventional display vector pecan126 whether in HB2151 or HB2151+pecan134. Since pecan134 and 114 do not express sdAb or biotinylated sdAb respectively they serve as negatives. Checker-board titration of tenfold dilutions of captor (x-axis) with tenfold dilutions of tracer (bars left to right of each set) using either *Marburgvirus* positive control antigen (left) or *Ebolavirus* negative control antigen.

We hypothesized that it should be possible for the g3p on one plasmid to display the sdAb on another plasmid (the *phagemid* to ensure rescue of the sdAb genotype) in *trans via* capture of the hapten biotin, if it were afforded a streptavidin (strep) motif as shown in the schematic (Fig. 4a) with the expression cassettes summarised in Figure 4b. We first tested all of the N-out C-in phage coat proteins for their ability to display a streptavidin minigene as an amber suppressed motif^38^ capable of binding biotin to reveal that full-length g3p was the superior display platform (Supplementary Fig. S8). We then amplified the strep-coat protein genes with a primer encoding supE-V88 and a common back primer and replaced the pSCUPER V88 *supE* gene with them and verified streptavidin display still occurred (Supplementary Fig. S9). To provide the source of biotinylated sdAb the g3p region of pecan 126 was exchanged for stop codons to create pecan 133, and co-transformed into HB2151 with the various supE-V88-streptavidin fusion vectors to reveal that only g3p appeared permissive for transdisplay under various IPTG/arabinose combinations (Supplementary Fig. S10). Finally, the optimal conditions were used in a comparison with pecan126 in XL1-Blue and HBV88 to reveal an approx. 10 fold drop in signal strength for pecan133+134 in HB2151 by phage monoclonal ELISA (Fig. 4c). However, rapid antibody pairing showed equivalent signals to the HBV88 system (compare Fig. 4d with Supplementary Fig. S7) since the soluble expression mechanics are the same between pecan126 and pecan 133 and phage display is excluded from the equation.

### From immune repertoire display to rapid antibody pairing in the new host/vector combinations

**Figure 5:**
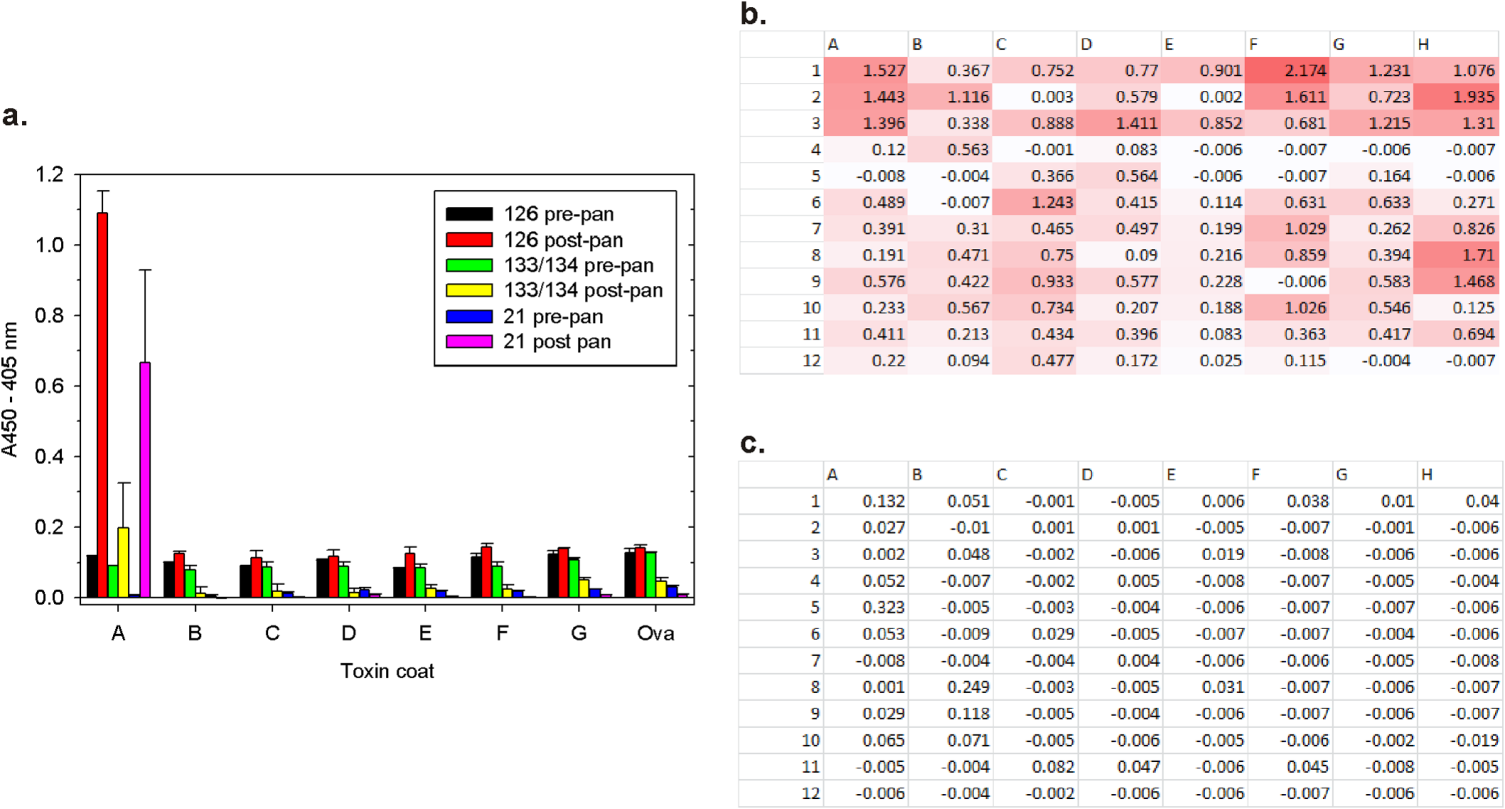
Deconvoluting a BoNT llama immune library selected *via* conventional sdAb-g3p fusion display and sdAb:g3p transdisplay. (a) Polyclonal phage ELISA pre- and post a single round of panning on BoNT A using a heptaplex immune library retrofitted for display in HBV88 (126) or transdisplay (133 134) vs the original conventional display route using XL1-Blue and a standard non-BAP gene III fusion vector (21). (b) Monoclonal phage ELISA of 96 pecan126 clones within HBV88 on BoNT A antigen (negative control antigen in Supplementary Fig. S11 showed no background signals). (c) Monoclonal phage ELISA of 96 pecan133 clones within HBP134 on BoNT A antigen (negative control antigen in Supplementary Fig. S12 showed no background signals).

**Figure 6:**
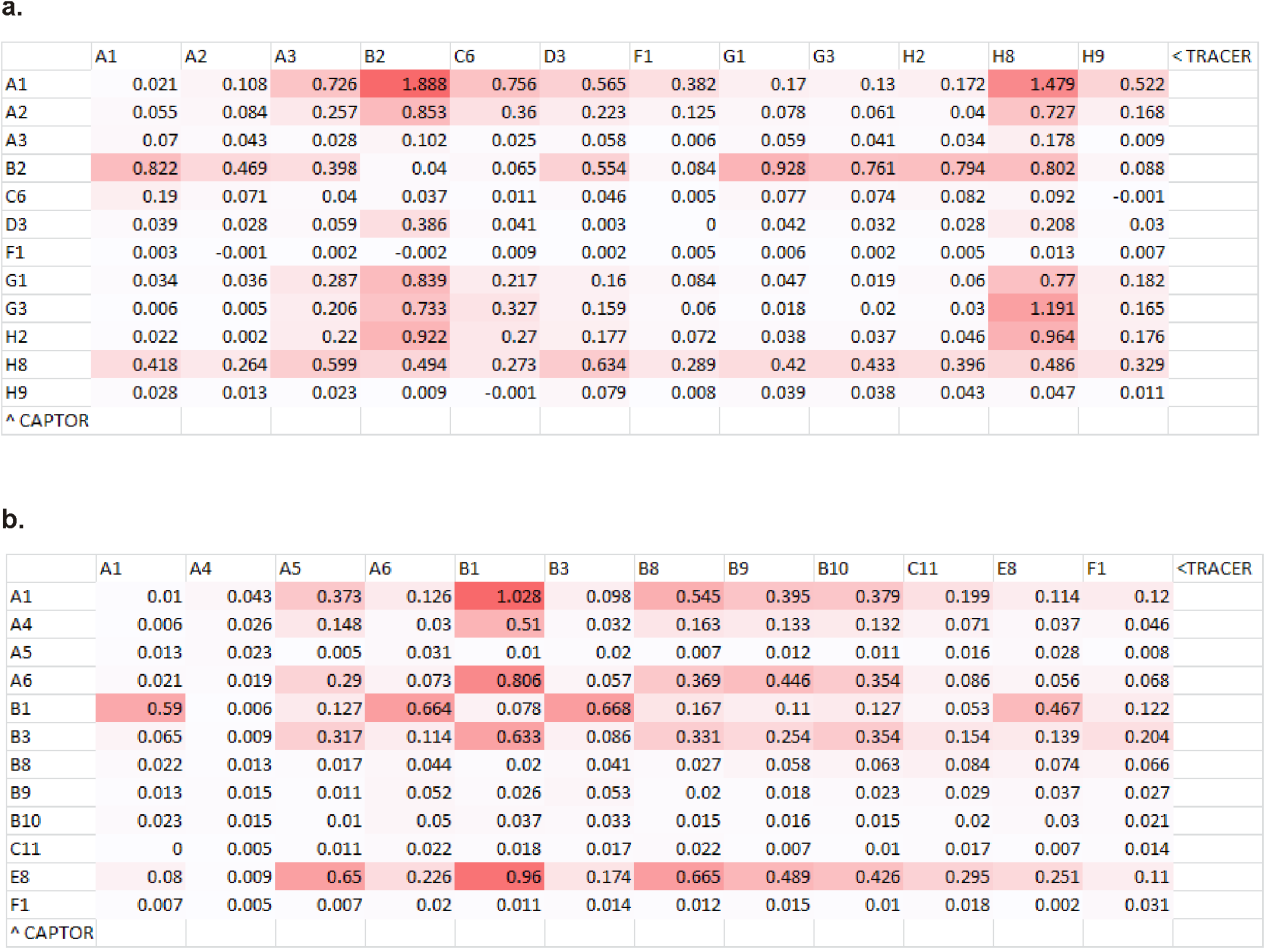
Applying rapid antibody pairing to the monoclonal anti-BoNT A sdAb clones identified through conventional phage display and transdisplay. (a) Pairing of sdAb clones identified from pecan126/HBV88 heptaplex BoNT immune library retrofitting, selection and screening reveals several promising combinations of sdAb capable of recognizing 1 µgmL^−1^ BoNT serotype A. Negative control antigen performed alongside showed no background signals (Supplementary Fig. S13). (b) Pairing of sdAb clones identified from pecan133/HBP134 heptaplex BoNT immune library retrofitting, selection and screening reveals several promising combinations of sdAb capable of recognizing 1 µgmL^−1^ BoNT serotype A. Negative control antigen performed alongside showed no background signals (Supplementary Fig. S14).

**Figure 7:**
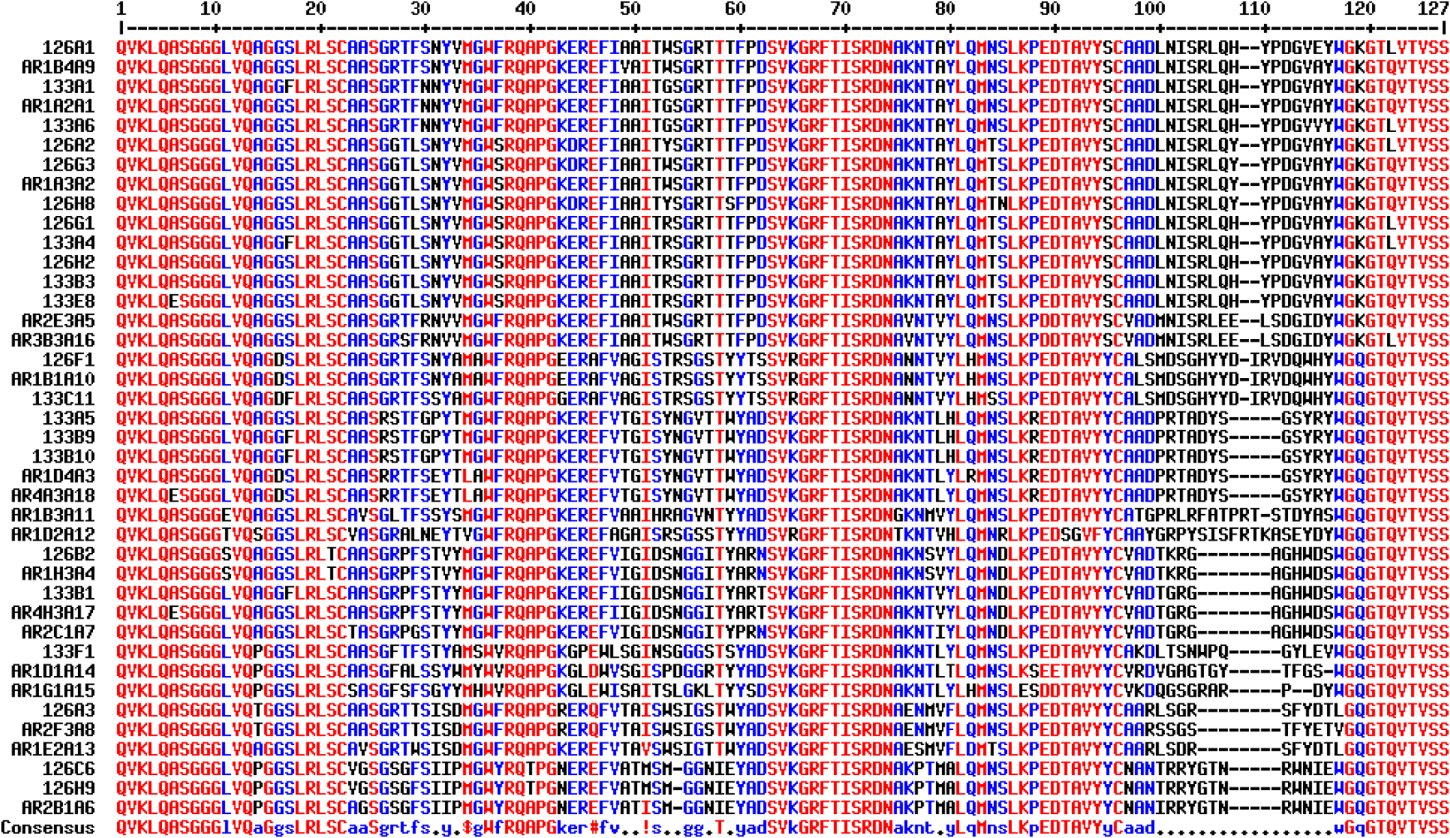
Sequencing the anti-BoNT A clones reveals similarity to those isolated previously using a non-BAP based / non-transdisplay system. Predicted amino acid sequences of sdAb clones selected using pecan126 within HBV88 (126), pecan133 within HBP134 (133) aligned with the original anti-BoNT A clones from conventional panning^3^ where A designates anti-A serotype, R designates round of isolation, XY denotes ELISA plate well, and An designates rename 1 through 18. Pecan 133 and pecan134 share the lacZ' priming site and must be sequenced with AHX76. Clone D3 from the pecan126 selection was not able to be fully sequenced, appearing to terminate before CDR3 and clone B8 from the pecan133 selection terminated before the sdAb gene. Sequences were aligned with Multalin^65^.

To demonstrate the dual use host HBV88 system was indeed useful for repertoire selection, the library originally used to derive the BoNT pairs was retrofitted with pecan126 to make a 3e+8 cfu library (approx. 1/3^rd^ of the original size), rescued within HBV88 and panned on immobilized BoNT A toxin for one round. Similarly, to demonstrate the transdisplay system could be useful in repertoire selection the original BoNT library was retrofitted into pecan 133 to make a 2e+9 cfu member library, (twice the size of the original) and also used to pan for a single round. Polyclonal ELISA was performed to show enrichment of BoNT A populations for conventional vector pecan21, pecan126 and pecan133 though the signals are very much weaker for the latter transdisplay format (Fig. 5a). 96 clones were picked from each system and screened as phage to reveal strongly positive clones for HBV88 (Fig. 5b) and apparently weaker ones for transdisplay (Fig. 5c) with equivalent negative control signals shown in Supplementary Fig. S11 and S12 respectively. The highest 12 signals from each system were picked and employed in soluble expression mode for checker-board rapid antibody pairing to reveal suitable sdAb combinations that bound solution phase toxin (Fig. 6a and 6b) with corresponding negative controls shown in Supplementary Fig. S13 and S14 respectively. Note that the transdisplay based vector signals are now on a par with HBV88 since phage display is excluded from the comparison. Only after pairing had occurred was sequencing performed to reveal the positive clones from each system were similar to those isolated previously using conventional methods (Fig. 7).

### From single pot library to rapid antibody pairing using transdisplay

**Figure 8:**
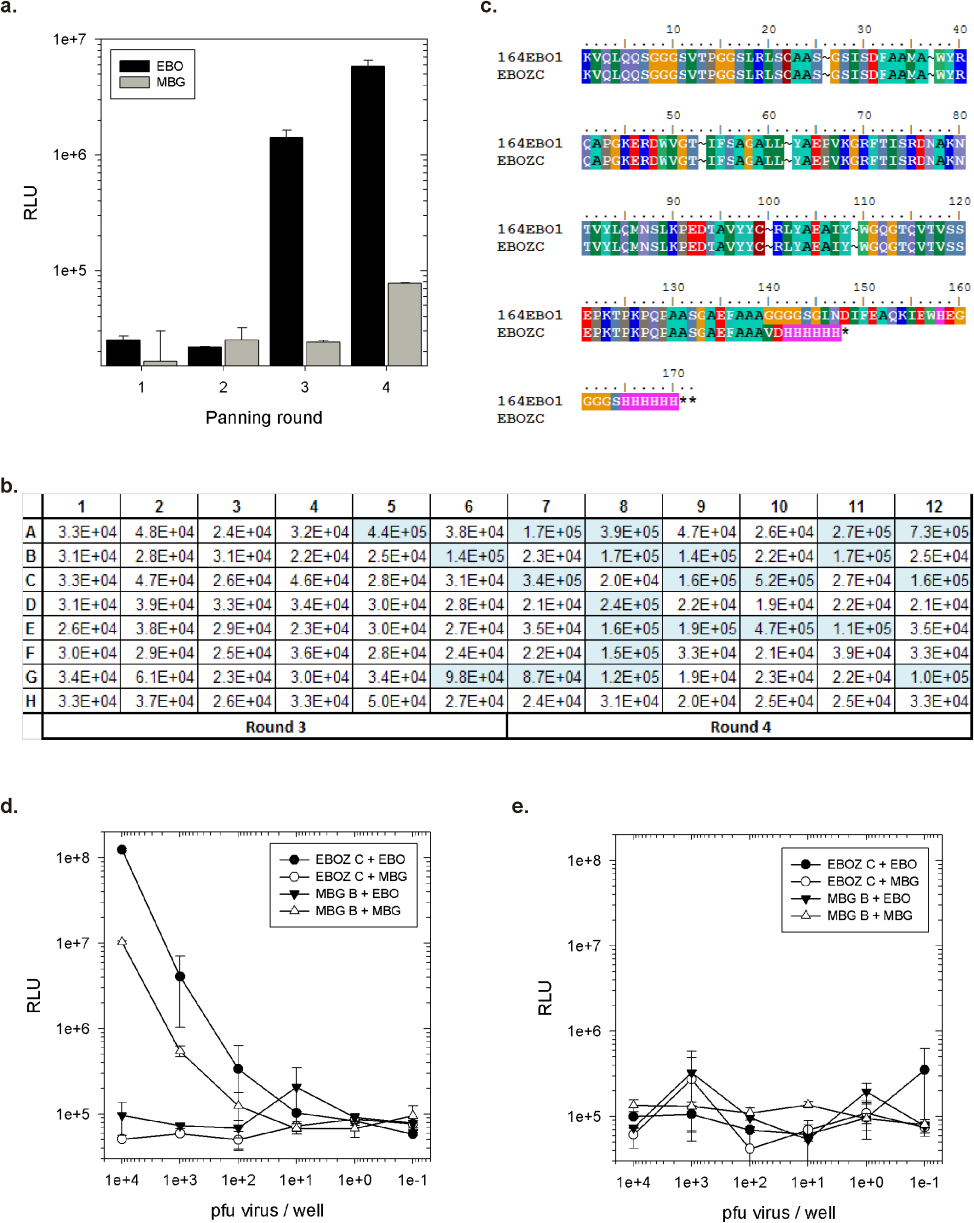
Using transdisplay to select anti-*Ebolavirus* Zaire sdAb from a retrofitted single pot sdAb library and yield an antigen capture assay without component purification. (a) Enrichment of polyclonal phage specific for *Ebolavirus* Zaire (EBO) rather than *Marburgvirus* Musoke (MBG) through four rounds of panning the semi-synthetic llama sdAb library Nomad#1 within HygR pecan133 (pecan164) in HBP134 on *Ebolavirus* Zaire virus. (b) Monoclonal phage ELISA of 48 clones from round 3 and 4 on Zaire target and Musoke control viruses to identify the potential positive clones for sequencing (corresponding negative control ELISA is shown in Supplementary Fig. S15). (c) Predicted amino acid sequence of the single clone identified from 22/22 sequences and comparison with the original isolate in a non-BAP display vector. (d) The single sdAb clone (EBOZ C) borne on pecan164 within HBP134 was produced as osmotic shockate for captor and phage as tracer to generate an *Ebolavirus* Zaire (EBO) specific assay, while MBGB captor and phage generated from HBP134 carrying pecan133MBGB served to confirm *Marburgvirus* (MBG) control was present. (e) Performed side by side with the phage tracer experiment in fig 8d on the very same dilutions of virus we find using sdAb as both captor and tracer reveals that at these low virus numbers the sdAb-sdAb pairing system can struggle for sensitivity without phage amplification.

We next sought to apply transdisplay and pairing to a single pot library to determine if we could truly accelerate immunoassay formulation through bypassing immunization, avoiding antibody purification and rapidly pairing antibodies. Since our semi-synthetic llama sdAb library “Nomad#1”^39^ had proven successful in generating a specific and sensitive sdAb to both *Marburgvirus*^18^ and *Ebolavirus*^27^ nucleoproteins using conventional sdAb-g3p panning we retrofitted it for transdisplay to compare and contrast clones selected using the new approach. The library was made in a hygromycin resistant version of pecan133 (pecan164) to eliminate possible enrichment of existing sdAb in ampicillin display vectors and then selected on *Ebolavirus* Zaire (strain Kikwit 1995) at BSL-4. Polyclonal phage ELISA showed enrichment of an *Ebolavirus* specific population (Fig. 8a). Monoclonal phage ELISA potentially revealed 3/48 positive clones from round 3 and 20/48 positive clones from round 4 (Fig. 8b) with the corresponding negative control ELISA shown in Supplementary Fig. S15. The positives were sequenced to reveal 1 failed sequence and 22 clones matching a clone (EBOZ C) that we had isolated previously at a frequency of 11/24 DNA sequences (predicted amino acid sequences shown in Fig. 8c). Importantly, EBOZ C had never been individually mobilized to any pairing, transdisplay or hygromycin based vector and could only have arisen through enrichment from the retrofitted single-pot library. Previously, conventional panning had yielded EBOZ C and 6 other unique anti-*Ebolavirus* Zaire sdAb sequences from 24 sequenced clones (7 from round 3 and 17 from round 4) occurring 3, 3, 2, 1, 3 and 1 time indicating that we had only managed to isolate the most popular clone. This limited response could be due to their absence in the retrofit of the single-pot library since it only matched the original library size and should ideally be several-fold larger to represent multiple clones of each starting sdAb. Furthermore, we are already aware from Fig. 4c that transdisplay is currently around a log less effective at displaying sdAb than direct display, and will require optimization to be on a par with conventional phage display.

Despite these reservations, the EBOZ C clone was directly employed *via* the dual use host HB2151+pecan134 in a sdAb captor/phage tracer Filovirus titration (Fig. 8d) and a matching sdAb captor/sdAb tracer pairing (Fig. 8e) using small-scale osmotic shocks as before. The phage tracer yielded a relatively sensitive assay capable of detecting hundreds of pfu, the key to assay sensitivity likely relying upon the sdAb binding polyvalent nucleocapsid assemblies remaining intact after detergent treatment^40^ to yield a highly avid antigen format. However, the sdAb pairing struggled at these low antigen concentrations essentially yielding negative data reflecting the decrease in signals visualized on surrogate antigens previously for MBG B sdAb in Figures 2b and 2c without the amplifying effect of the phage and anti-phage conjugate. Despite this caveat, using transdisplay we have succeeded in demonstrating proof of principle in transitioning from a single pot library to an antigen capture assay without antibody purification albeit employing phage tracer at the moment. Further optimization of the transdisplay system is underway to enable higher library assembly and display efficiencies to enable us to recover all of the clones residing in the original single-pot library. Additionally, access to higher quality libraries^41^ and diversity while retaining solubility^42^ or assembly of a larger sdAb repertoire based upon more donors all may well yield a broader diversity of more sensitive clones for immediate use on low biothreat virus numbers with just antibody pairing alone without phage tracing.

## Discussion

Our goal throughout was to develop a simple technology that accelerates the generation, characterization and utilization of recombinant antibodies as sandwich assay components from repertoires. While our main focus was to improve the delivery of stop-gap assays to biothreat agents we see no reason why these methods cannot be used to streamline assay formulation to any target of interest including non-infectious agents. The only requirement for rapid antibody pairing we envisage is that the chosen antigen would need to be large enough or multimeric to enable at least two antibodies to bind non-competitively. Though the method may therefore not appear immediately suitable for small molecules which typically require competitive/displacement style assay formulations, it may well indeed prove useful for quickly screening pairs to establish open sandwich assays^43^. By using a single host to deliver site specific biotinylated antibodies from repertoires the phage display process becomes more suitable for resource limited laboratories in reducing the overall costs of the process and avoids subcloning a panel of antibody genes into specialized vectors and other hosts. The small scale osmotic shockate recombinant antibody preparations may also find use as initial screening probes for western blots, flow cytometry, microscopy, etc. to further refine their specificities, though this will have to be validated in further experimentation. Overall however, the approach should also smooth the pathway to robotic antibody pairing if available and checker-boarding using chip^14^ or fluid based array technologies to build upon automation of panning and screening processes^44^,^45^ with the affinity reagents immediately biosensor ready^46^. Although we prefer to use single domain antibodies owing to their plasticity^47^, broad applicability^48^ including viral cryptic epitope targeting^49^, the rapid pairing approach should be applicable to other recombinant antibody formats^1^, alternative scaffolds^50^ and smaller motifs (peptides, aptamers etc.) and assemblies^51^. Furthermore, though we use phage display owing to its simplicity and capacity to handle multiple representations of large repertoires, we envisage transdisplay could concomitantly be incorporated in any platform where the biotinylated motif and an anchored streptavidin can be transiently co-compartmentalized without the motif leaching excessively e.g. APEX^52^, MAD-TRAP^53^ etc.

## Methods

### General

Recombinant DNA methods were according to established procedures and employed commercially available reagents; HotStart YieldAce, (Stratagene, La Jolla, CA) was used for PCR amplification unless otherwise noted; restriction enzymes and β-agarase (New England BioLabs, Beverly, MA); T4 DNA ligase, CIP and T4 PNK (Roche, Nutley, NJ), AgarACE (Promega, Madison, WI); GTG low melting temperature agarose, (Lonza, Walkersville, MD); oligonucleotides (Integrated DNA Technologies, Coralville, IA); synthetic core streptavidin gene (Genscript, Piscataway, NJ). Assemblies involving PCR amplification or oligonucleotide bridging were sequenced through the inserts and junctions to verify the desired construct. Cloning was typically in XL1-Blue unless otherwise stated, with constructs under study mobilized to hosts under investigation. Expression/display cultures were not supplemented with additional biotin.

### Biosafety

Select agent work was carried out at Texas Biomedical Research Institute following all federal guidelines as part of the CDC Select Agent Program and with local Biohazard and Safety Committee approval. Botulinum neurotoxins were handled in enhanced BSL-2 conditions while Filoviruses were handled in the full suit BSL-4 laboratory.

### Construction of expression and display vectors

Details may be found in the supplementary information accompanying this manuscript.

### Expression of sdAb

Small-scale cultures of 20 mL terrific broth (no glucose plus appropriate antibiotics: ampicillin 200 µgmL^−1^ for most pecan vectors, 200 µgmL^−1^ hygromycin B for pecan 164, 30 µgmL^−1^ chloramphenicol for pecan 134) were seeded with 400 µL of a saturated overnight 3.5 mL culture, grown with vigorous aeration for 2h at 30°C, 0.5 h at 25°C, induced with addition of IPTG to 1 mM and shaken for a further 3 h. Cells equivalent to approx. 20 OD at A600 nm cm^−1^ were harvested as described previously^54^ using a scaled down version of the Neu and Heppel method^55^, to yield approx. 1 mL volume of osmotic shockates, which were either used immediately or stored at −20°C until required. Mid-scale expression with subsequent IMAC and gel filtration was performed on 400 mL scale cultures as described previously^3^.

### Mass spectrometry

Kindly performed by Kevin Hakala and Dr. Susan Weintraub at the Institutional Mass Spectrometry Laboratory, UT Health Science Center San Antonio- ESI Infusion on the ThermoFinnigan Quantum Triple Quadrupole Mass Spectrometer – details within the supplementary information

### Antigens

Details on the assembly and purification of antigens used may be found in the supplementary information.

### ELISA pairing of sdAb

100 µL of neutravidin (Pierce, Rockford, IL) at 1 µgmL^−1^ in PBS was used to coat a 96 well high binding ELISA plate, either clear #3590 or white #3922 (Corning Costar, Tewksbury, MA) overnight at 4°C. The plate was washed 3 times with PBS, and blocked by addition of 375 µL of PBS + 2% BSA + 0.1% Tween-20 (PBSBT) for minimum of 1 h at room temperature. The block was replaced by 100 µL of fresh block containing the desired volume of shockate, typically 1 µL or 10 µLand left either o/n at 4°C or shaking (Barnstead Lab-Line plate shaker setting #2) at room temperature for 30 min to enable the neutravidin to capture the sdAb. The plate was washed 3x with 175 µL of PBS + 0.1% Tween-20 and 2x with PBS. 100 µL volumes of antigen were added in PBS + 2% non-fat dried Carnation milk (MPBS) and the plate shaken for 15–30 min. The plate was washed as before, the tracer (typically 1 µL of shockate) in 100 µL volume of PBSBT was added and the plates shaken for 15–30 min. The plate was washed as before and 100 µL of high sensitivity neutravidin HRP conjugate (Pierce) at 1 in 10,000 dilution in PBSBT was added, and the plate shaken for a further 15–30 min. The plate was washed as before and 100 µL of TMB-ultra (Pierce) added and stopped with 50 µL of 0.5 M H_2_SO_4_ typically after approx. 3 minutes, or the plate was developed with Pico ELISA chemiluminescent substrate (Pierce) and read on a luminometer (Turner Biosystems) with a 2s integration. The dilutions for titrations were performed in duplicate wells with the average plotted between maximum and minimum points. Each of these titrations was performed twice (Fig. 2b [phage tracer], 2c, 3c, 8d [phage tracer], 8e) and though the absolute ELISA readings varied as the incubation times were slightly different, the same trends were observed. Checkerboard titrations only represent single data points for each captor and tracer combination (Fig. 4d, 6a, 6b).

### Phage display and ELISA

For conventional display and vector proving, 400 µL of overnight cultures of phagemids in XL-1Blue/DH10-tetF' were used to inoculate 40 mL 2xTY 2% glucose pus appropriate antibiotics in 250 mL baffled flasks and shaken for approx. 2 h at 37°C until an O.D. of 0.4–0.6 was reached. M13K07 at an moi of 20 was mixed in, the culture left static for 30 min, and then shaken overnight at 30°C in the presence of 70 µgmL^−1^ kanamycin and 10 µM IPTG. Display using HBV88 employed 10 µM IPTG and 2000 µgmL^−1^ L-arabinose while display in HB2151+pecan134 utilized 10 µM IPTG and 200 µgmL^−1^ L-arabinose. 2 mL aliquots were clarified by centrifugation (13,500 rpm, 10 min) and stored at −20°C until required. Detection of phage in sdAb captor or direct phage ELISA on antigens was using standard methods and anti-M13-HRP conjugate. Phage ELISAs were generally performed in MPBS except the capture of strep-phage on biotinylated sdAb which was in PBSBT to avoid competition from biotin in milk. Phage titration experiments were duplicate ELISA plate wells of each dilution with plots representing the average reading with maximum and minimum values shown (Fig. 3b, 4c) with each ELISA performed twice and though the exact numbers varied as incubation times were different, the same trends and relative shifts were apparent. Polyclonal enrichment ELISAs represent the average of duplicate wells and were performed only once (Fig. 5a, 8a). Monoclonal phage ELISAs (Fig. 5b, 5c, 8b) were performed once.

### Library retrofitting

The previously assembled single-pot^39^ and immune library^3^ were stored as both phage and glycerol stocks, the latter containing enough representations to seed 6 x 400 mL cultures to an OD of 0.1 for re-rescue, ie. approx. 1.2 e+11 cfu. One 2 mL glycerol was made to 24 mL with media and divided into 12 x 2 mL and miniprepped (Qiagen) for elution of approx. 12 x 10 µg in 80 µL of EB. The destination vectors pecan126, pecan133 and pecan 164 were modified by replacement of the MBG B sdAb with a 2 kbp tet stuffer from pecan 21 via *Nco*I (partial)/ *Not*I to provide a convenient marker for scoring positive sdAb gene inserts and to enable *Sfi*I/*Sfi*I cloning^21^ for the single-pot retrofit while immune libraries employed *Sfi*I/*Not*I. Each vector was grown in 12 x 3.5 mL of terrific broth, 2% glucose and appropriate antibiotics and 12 x 2 mL miniprepped for elution of approx. 12 x 10 µg in 80 µL EB which were then pooled. Vectors and inserts were digested by addition of 120 µl 10 x React2 buffer, 120 µL 10 x BSA and 24 µL of *Sfi*I (20 U/µL) and left for 12–18 h in a 50°C oven. The immune library inserts and vectors were further digested by addition of 60 µL React 3 and 24 µL *Not*I (10 U/µL) for a further 12 h at 37°C. Vector digests were CIPped by addition of 120 µL 10xCIP buffer and 40 µL CIP (1 U/µL) and left at 37°C for 2 h (although self-ligation is not an issue here, we dephosphorylate to reduce inter vector-vector ligation reducing the available destination DNA for the insert). DNA was electrophoresed on 1% (for insert) or 0.5% (for vector) GTG TAE gels and the appropriate bands excised in a volume of about 2.5–3.5 mL for a scaled-up version of in agarose ligation^56^. The gels were melted at 70°C then kept at 37°C and both vector and insert were added and mixed in 400 µL aliquots into 6–8 tubes each containing 780 µL water, 200 µL 10xT4 DNA ligase buffer (Promega) and 20 µL β-Agarase (1U/µL) which stayed liquid at room temperature (approx. 75F). 20 µL of T4 DNA ligase (1 U/µL) was added, the tubes mixed gently by inversion, covered in foil and left at slightly warmer than room temperature atop the hybridization oven for 18–24 h. The ligations were aliquoted to 24–32 x 500 µL, each extracted with 450 µL of phenol/chloroform (Invitrogen), and 450 µL supernatants precipitated by addition of sodium acetate/ethanol and left on the bench for 2 h only to avoid excessive salt precipitation. Tubes were microfuged at 13.5 krpm for 15 min, supernatants poured off, pellets briefly washed with 350 µL 70% EtOH, recentrifuged for 5 min, aspirated and dried briefly in a tissue culture hood. Each pellet was resuspended in 42–32 µL (depending on the number of ligations) and pooled to provide a combined 32 aliquots of 60 µL for bulk electroporation which were aliquoted for storage at −80°C until required.

HBV88 or HB2151+pecan134 were made electrocompetent using low temperature growth^57^ in low salt YENB media^58^ growth followed by extensive washing^59^ and reliably yielded transformation efficiencies in the mid 1e+9 cfu/µg ccc pUC19 range only some 2–4 fold less than home-made preparations of high efficiency strains such as DH10B or DH10F'tet. A streak on M9/chloramphenicol + thiamine minimal agar to select the F'-episome was rinsed into 400 mL of liquid M9 equivalent and shaken overnight at 37°C. From this starter, 6x400 mL flasks of YENB plus chloramphenicol were inoculated to an OD600 nm of 0.05 cm^−1^ and shaken at 25°C until an OD of between 0.4 and 0.5 was reached (approx. 6–7 h). Cells were immediately pelleted (Allegra GPR, 4x750 mL pots, swing out rotor, 4°C, 20 min), resuspended gently in a total volume of approx. 1.5 L ice cold water and re-pelleted (Allegra, 6x250 mL, fixed angle, 5.75 krpm, 20 min 4°C). Cells were washed in water once more, then washed in 15% glycerol and finally resuspended in 8.7 mL of 15% glycerol and aliquoted to 32 x 270 µL, snap frozen in −80°C isopropanol and stored frozen until required.

Electroporations of the 60 µL ligation and 270 µL cell aliquots were performed using an electroporator (BioRad) at 2.5 kV with 2 mm gap electrocuvettes (Bulldog Bio Inc., Portsmouth, NH) that accommodated the combined volume. Following electroporation the cuvette contents were poured into 50 mL Falcon tubes followed by 3 cuvette washes of 2 mL of prewarmed (37°C) SOB 2% glucose with two electroporations worth pooled in each Falcon tube. The mixes were left static for 60 min and cells were then pelleted (Allegra GPR, 3 krpm, 10 min, 20°C), resuspended in 400 µL supernatant and spread on Bioassay dishes containing 250 mL 2xTY 2% glucose with appropriate antibiotics. Following growth overnight, cells were scraped with a 3” wallpaper scraper (Hyde, Home Depot, San Antonio) to a pot and the plates also washed with 4 mL of 2xTY 2% glucose which was added to the pot and the whole lot mixed thoroughly. After measuring the OD600nm*, 6 flasks of 400 mL 2xTY 2% glucose were seeded to and OD600nm of 0.05, grown with shaking at 37°C to an OD of 0.4–0.5 and infected with M13K07 at an moi of 20 for 1 h static. Kanamycin, IPTG and arabinose was added according to the system employed and rescue proceeded for 18–24 h at 30°C. Cultures were clarified by centrifugation (Sorvall RC6+, 4x1 L, 8 krpm, 1 h, 4°C), pooled and precipitated by addition of 480 mL of 20% PEG6000/2.5 M NaCl and overnight stirring at 4°C. Phagemids were pelleted (Allegra GPR, 3.75 krpm, 4x750 mL swing out, 1 h, 4°C), drained and resuspended in a total volume of 16 mL PBS to which was added 16 mL of glycerol, and 16 x 2 mL aliquots stored at −80°C until required. *The remainder of the cell suspension was combined with an equal volume of ice cold 30% glycerol in terrific broth and aliquoted into 40–50 2 mL cryovials such that each aliquot could seed another six flasks if required.

### Library selections

Conventional panning methods were used for these targets^2^. An 8 well strip was coated with 8x100 µL of antigen overnight at 4°C. Wells were washed 3x with 175 µL PBS and blocked for 1 h with 350 µL MPBS. Phage representing 100 clones of each library were applied in 8x100 µL MBPS for 30 min shaking, wells washed with PBST and PBS before 8x100 µL triethylamine elution for 10 min followed by pooling and neutralization with 400 µL 1M Tris-HCl pH 7.5. For BoNT A, the single round of panning was 20 washes each and for *Ebolavirus* Zaire it was 10, 20, 20, 30 each for rounds 1 through 4 respectively. 600 µL of neutralized eluate was added to 10 mL of mid exponential phase HBV88 or HB1251+pecan134 as appropriate for 30 min before titrating an aliquot while the rest was gently pelleted and plated on 15 cm diameter dishes. Overnight growth was followed by scraping large plates for glycerol stocking and liquid culture for M13K07 superinfection and display. Induction conditions are described above in phage display and ELISA. Polyclonal ELISAs utilized aliquots of the saved superinfected supernatants while monoclonal ELISAs were derived from the titration plates.

## Author Contributions

AH conceived, designed and performed experiments to establish the pairing, dual use host and transdisplay systems, AH and LJS performed conventional and transdisplay selections on live virus and LJS characterized the sdAb isolated. LJS and AH analyzed data. AH wrote the paper.

## Supplementary Material

Supplementary Information Supplementary information

## Figures and Tables

**Figure 1:**
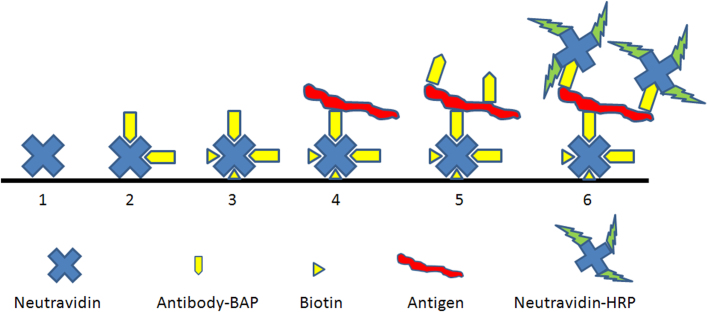
Schematic of how rapid antibody pairing *via* temporal occlusion of site specific biotinylation works. 1. Neutravidin is coated on a surface, herein passively absorbed onto wells of an ELISA plate; 2. Crude osmotic shockate is applied to the system and antibody is captured *via* the single biotin moiety; 3. Unoccupied biotin binding sites are blocked with free biotin; 4. Antigen is added and is captured by the immobilized sdAb (steps 3 and 4 are typically concomitant since antigen is applied in 2% non-fat milk which contains biotin); 5. Crude osmotic shockate is again applied and polyvalent antigen captures sdAb tracer; 6. Neutravidin charged with horseradish peroxidase is added which can only bind to that sdAb that has unoccluded biotin (ie. tracer), and substrate is added for signal development.
