# Supplementary material for: Hapten Mediated Display and Pairing of Recombinant Antibodies Accelerates Assay Assembly for Biothreat Countermeasures
Source: Sci Rep. 2012 Nov 12;2:807. doi: 10.1038/srep00807 (PMC3495282; doi:10.1038/srep00807)
Supplement: Supplementary Information [file srep00807-s1.pdf]

Supplementary Data for "Hapten Mediated Display and Pairing of Recombinant Antibodies Accelerates Assay Assembly for Biothreat Countermeasures" Laura J. Sherwood and Andrew Hayhurst

Figure S1. Recombinant nucleoprotein antigens enable convenient path-finding at BSL-2

Demonstrating recombinant Marburg and Ebola Zaire virus NP mimics are polyvalent within crude cytosolic extracts of *E. coli* and therefore acceptable surrogates for path finding the pairing system at biosafety level 2 (BSL-2). Clarified lysates of Tuner bearing pecan42 driving the tac based expression of tagless Marburgvirus Musoke (MBG) NP or Ebolavirus Zaire Kikwit (EBO) NP genes were titrated over duplicate wells coated with 100 µL 100 nM unbiotinylated sdAb derived from pecan45. EBOZ C was a clone selected on Zaire virus and MBG C was a clone selected on Marburg virus using standard display methods. Antigen capture was detected with 100 µL of 100 nM sdAb-AP fusion and LumiPhos chemiluminescent detection.

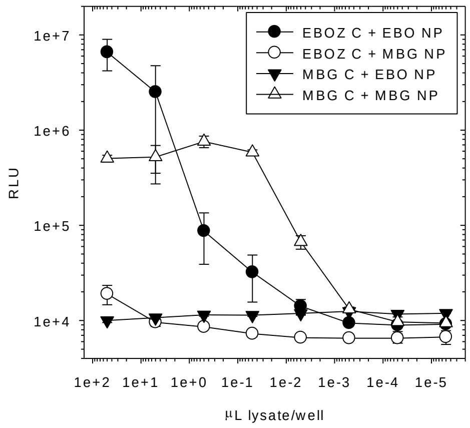

Figure S2. Conflicts of interest between expression cassettes and propensity to be phage displayed

Phage display ELISA of initial pairing vectors and linker variant vectors in XL1-Blue and HBV88 reveals potential conflict between full length birA (pecan 123 and 124) especially when initiated by ATG (pecan123). pecan130, 131 and 132 encode sdAb-BAP-His6, sdAb-G4S-BAP-His6, and sdAb-BAP-G4S-His6 fusions to g3p respectively. Capture antigen was purified Marburg Musoke NP-His6 protein. The lowest dilution used (1 in 2) was used to probe duplicate wells of Ebola Zaire NP-His6 protein yielding signals from 0.001 to -0.006 (data not shown).

XL-1 Blue, primary vector set

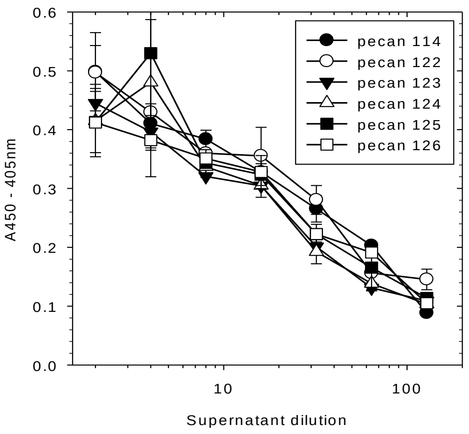

HBV88, primary vector set

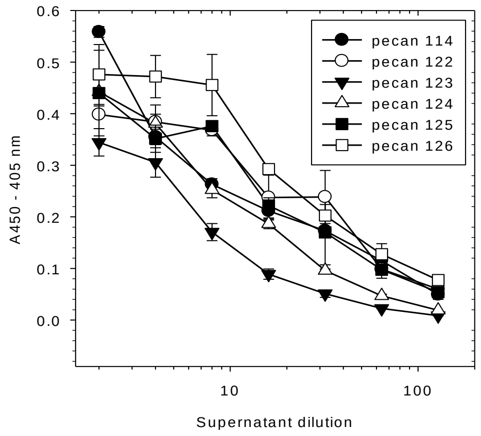

XL-1 Blue, linker variant set

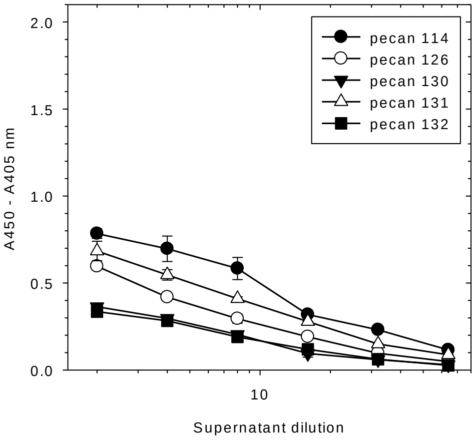

HBV88, linker variant set

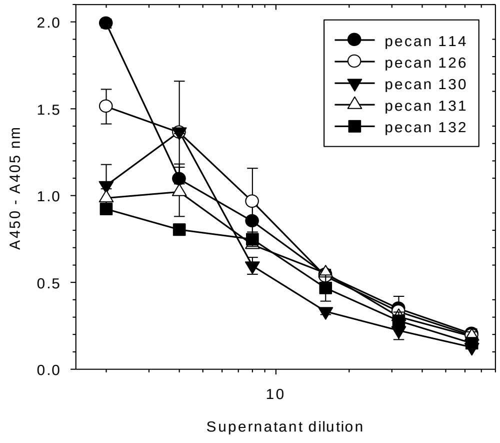

Figure S3. Comparison of inducible *supE* vehicles in a strain other than HB2151.

The highly electrocompetent strain DH10B was conjugated with XL1-Blue and progeny selected on streptomycin and tetracycline to make DH10F'tet, a host suitable for phage display trials. DH10F'tet bearing pecan 114 and the supE plasmids were superinfected with M13K07 and induced with 10 µM IPTG and supernatants analyzed for capture by recombinant *Marburgvirus* NP. Control signals on *Ebolavirus* NP were -0.010 to -0.002.

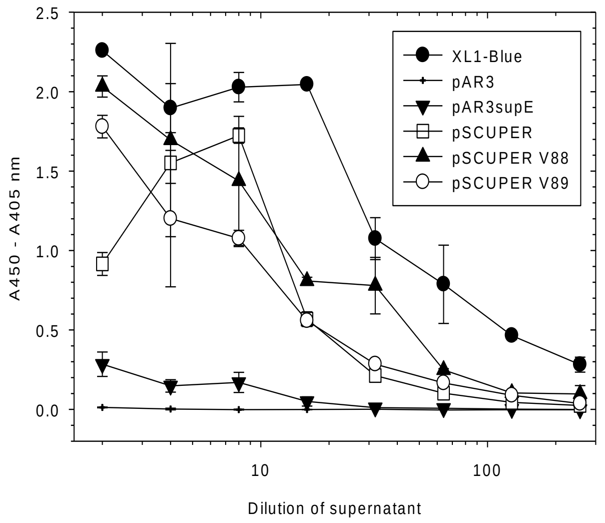

Figure S4. Impact of linkers flanking the BAP on soluble protein expression.

Gel filtration UV chromatograms of IMAC purified linker variants expressed within HB2151 and HBV88 in soluble sdAb production mode from equivalent wet weights of cells showing the parental pecan126 is a superior producer among the BAP tag class should scale up be required. Typical 400 mL shake flask cultures reliably yield approx. 15 mg of highly pure sdAb from pecan 126.

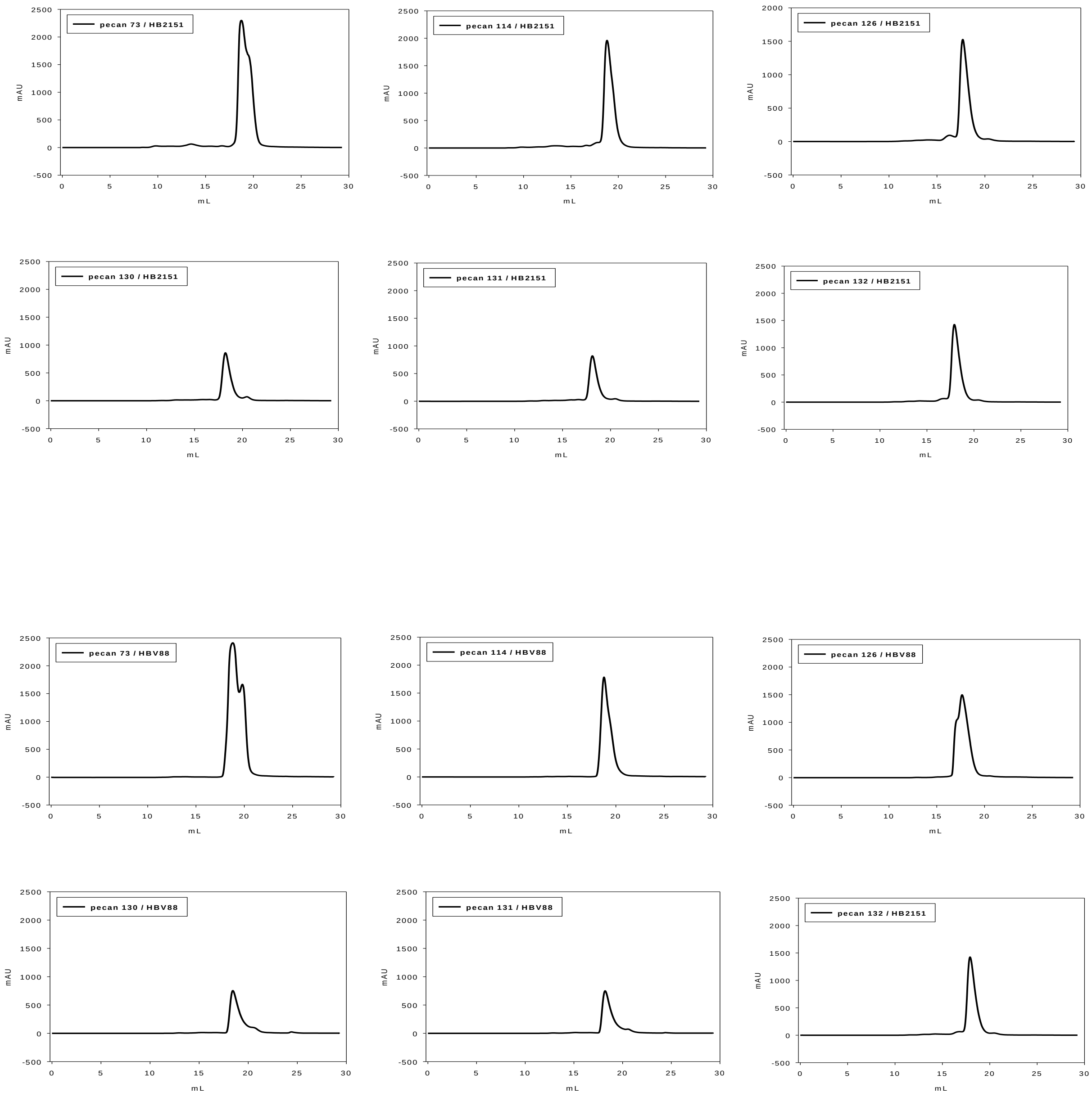

Figure S5. Purity of sdAb from linker variants in inducible supE strain versus parent.

Coomassie stained major peak fractions from HB2151 and HBV88 mid-scale preps of the linker variant vectors to show equivalent extents of purification from each of the vectors afforded by standard IMAC and gel filtration. 8 µL of the peak and flanking fractions were combined with 32 µL of water and 40 µL of Laemmli reducing sample buffer, boiled for 3 min, cooled and 20 µL of each sample was loaded on 15% Laemmli SDS-PAGE. M represents unstained molecular weight markers (Biorad) in kDa.

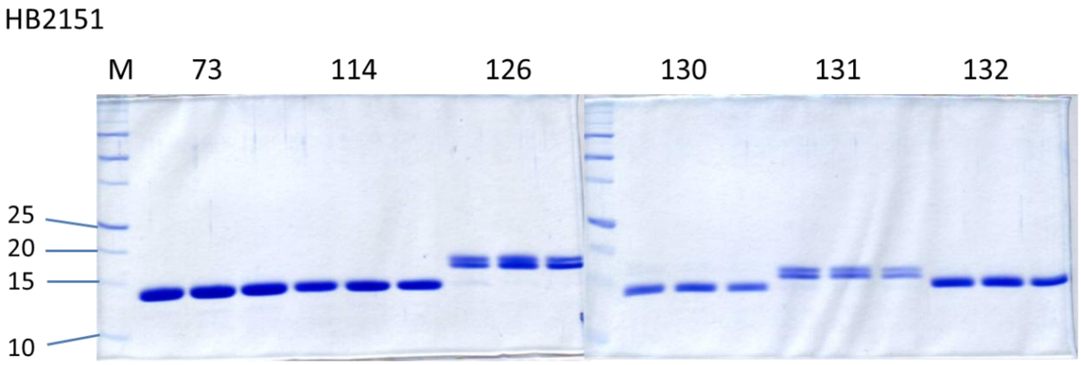

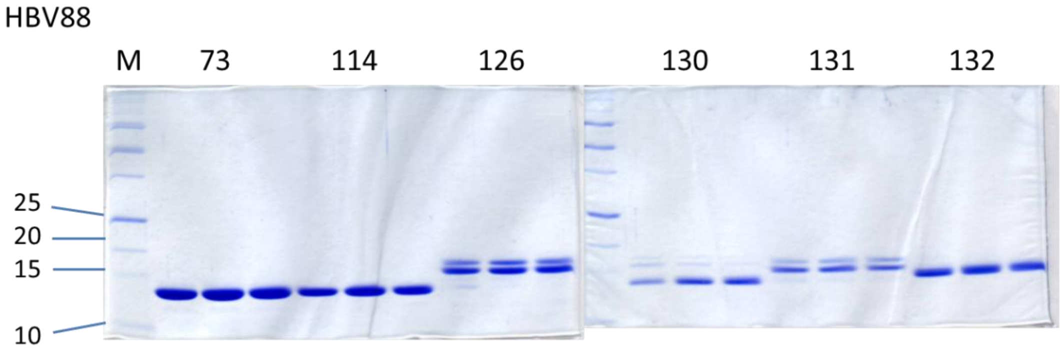

Figure S6. Ratios of biotinylated and unbiotinylated sdAb in purified proteins

Mass spectrometric analysis of the central peak fractions of pure sdAb preparations to discriminate between biotinylated and unbiotinylated protein in the linker variants indicates the doublets may be a consequence of BAP secondary structure when the predicted masses are; pecan73 and pecan114, 13,837 Da; pecan126, 16,278 + 226 Da; pecan 130, 15,648 + 226 Da; pecan131, 15,963 + 226 Da; pecan 132, 15,963 + 226 Da.

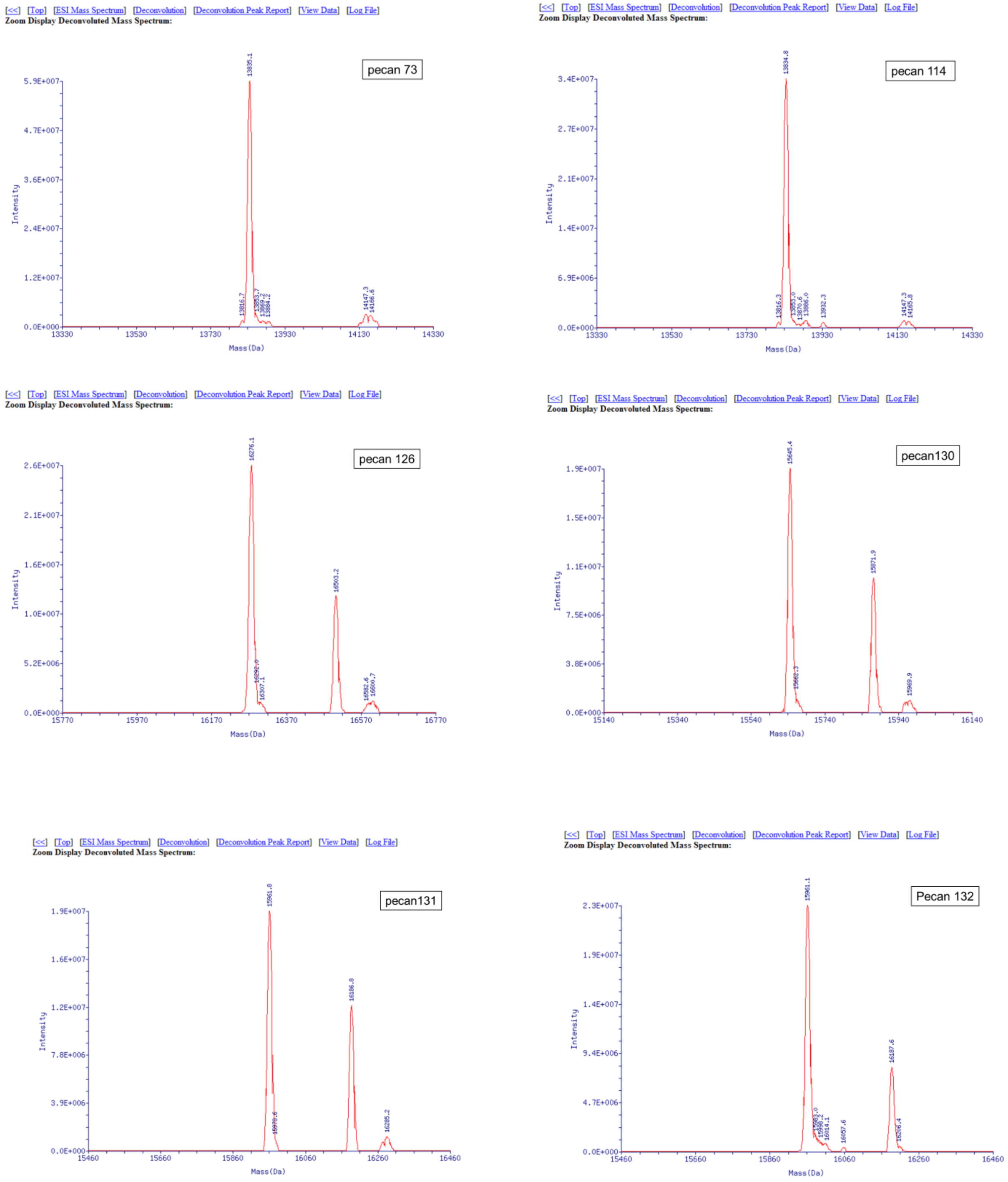

Figure S7. Rapid antibody pairing applied to linker variants of anti-*Marburgvirus* NP sdAb

Pairing of small-scale shockates from the linker variant phagemids in HBV88 by chequerboard titration of tenfold dilutions of captor (x-axis) with tenfold dilutions of tracer (bars left to right of each set) using either *Marburgvirus* positive control antigen (left) or *Ebolavirus* negative control antigen

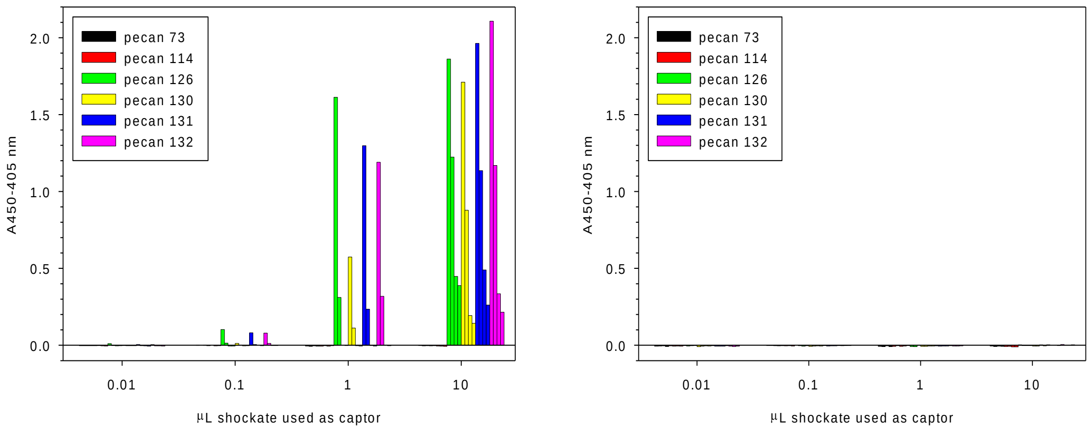

Figure S8. **Examining phage coat protein genes abilities to display sdAb and core streptavidin**

ELISA capture of sdAb (sdAb) or core streptavidin (STREP) displayed on the various coat proteins of M13 within a pecan114 backbone in XL-1 Blue to determine which platform to proceed with trans-display. The coating antigen was either Marburg NP protein or an equivalent amount of the purified sdAb-BAP generated from pecan 126 (**Supplementary Fig. 3c**) to show that all platforms appeared to display enough streptavidin to be specifically bound by biotinylated sdAb.

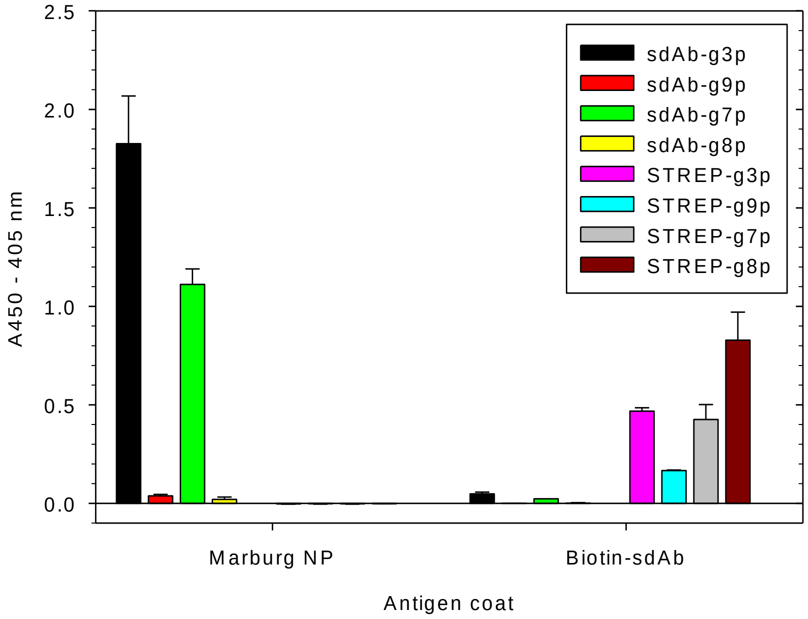

Figure S9. **Examining ability of supEV88 fused to core streptavidin-display protein fusion to enable display regardless of supEV88 production**

ELISA capture of pSCUPER backbone vectors having the supE exchanged for supE- streptavidin-platform genes, within XL-1 Blue (constitutively supE positive) initially to verify that the streptavidin is still sufficiently displayed when all but g9p is used, albeit weakly.

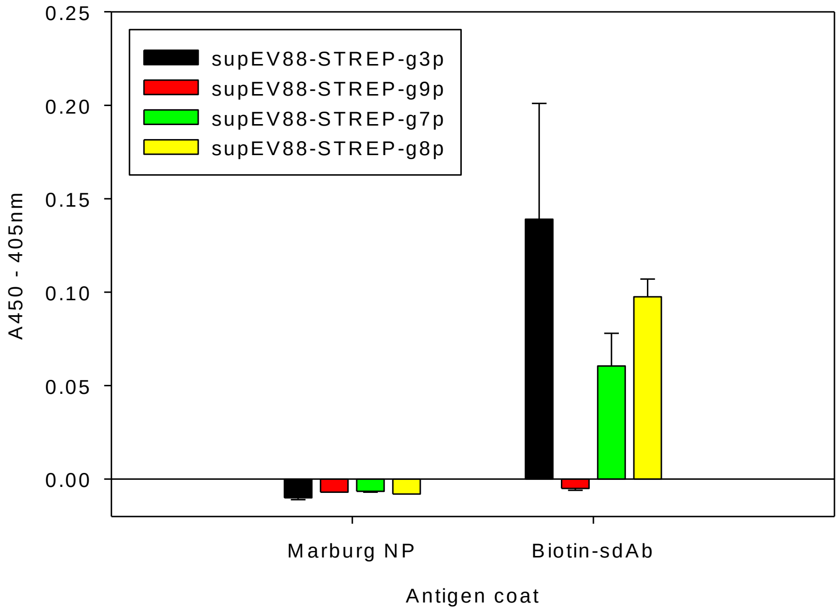

Figure S10. Proving functionality of supEV88 fusion with core streptavidin-fusion protein to enable transdisplay of sdAb

Single wells from a checker-board optimization of arabinose (2000 to 2 µgmL^{-1}) and IPTG concentrations (1000 to 1 µM) for transdisplay in HB2151 bearing supEV88-streptavidin platforms with pecan133. Vectors pecan134, 135, 136 and 137 encoded supEV8-streptavidin fusions to g3p, gp9, gp7 and gp8 respectively, while the control was empty HB2151. Only the g3p based vector had detectable signals at any combination of arabinose and IPTG.

		IPTG (mM)								
		MBG NP				EBO NP				
	Arabinose µg mL^{-1}	1	0.1	0.01	0.001	1	0.1	0.01	0.001	
	2000	0.032	0.058	0.002	-0.04	-0.002	-0.007	-0.006	-0.003	133/134
	200	0.007	0.055	0.256	0.077	-0.005	-0.009	-0.005	-0.004	133/134
	20	0.004	0.019	0.074	0.017	-0.002	0.001	0	0.002	133/134
	2	0.001	0.009	0.027	0.013	-0.012	-0.006	-0.003	-0.002	133/134
	2000	-0.006	-0.007	-0.006	-0.007	-0.007	-0.008	-0.007	-0.01	133/135
	200	-0.004	-0.006	-0.004	-0.005	-0.008	-0.006	-0.009	-0.008	133/135
	20	0	-0.003	-0.001	-0.004	-0.003	-0.004	-0.005	-0.007	133/135
	2	-0.004	-0.005	-0.002	-0.004	-0.004	-0.005	-0.006	-0.006	133/135
	2000	-0.004	-0.001	-0.002	0	-0.009	-0.007	-0.001	-0.006	133/136
	200	-0.007	-0.006	-0.002	-0.001	-0.006	-0.006	-0.002	-0.005	133/136
	20	-0.008	-0.004	-0.002	-0.004	-0.06	-0.003	-0.001	-0.005	133/136
	2	-0.006	-0.005	-0.003	-0.004	-0.005	-0.003	-0.001	-0.005	133/136
	2000	-0.001	-0.004	-0.001	-0.004	0	0.006	0.001	0.036	133/137
	200	0.014	-0.005	0.002	0.032	0.009	-0.07	0.003	0.051	133/137
	20	-0.005	-0.004	0.006	0.003	-0.006	-0.003	-0.001	0.014	133/137
	2	-0.005	-0.006	0.007	-0.001	-0.002	-0.005	-0.006	0.006	133/137
	2000	-0.006	-0.008	-0.009	-0.009	-0.012	-0.015	-0.017	-0.02	control
	200	-0.006	-0.005	-0.009	-0.002	-0.01	-0.012	-0.017	-0.013	control
	20	0	-0.028	-0.005	-0.006	-0.009	-0.01	-0.011	-0.015	control
	2	-0.002	-0.003	-0.004	-0.003	-0.007	-0.008	-0.006	-0.009	control

Figure S11. **Negative control monoclonal phage ELISA for pecan126 anti-BoNT A clones**

Monoclonal phage ELISA of 96 round 1 clones from HBV88 + pecan126 immune heptaplex library screened on 1 μgmL^{-1} of control antigen ovalbumin.

	A	B	C	D	E	F	G	H
1	-0.005	-0.007	-0.001	-0.007	-0.008	0	-0.01	-0.007
2	-0.007	-0.008	-0.001	-0.005	-0.004	-0.006	-0.004	-0.007
3	-0.008	-0.006	-0.005	-0.007	-0.006	-0.006	-0.004	-0.005
4	-0.006	-0.008	-0.001	-0.006	-0.006	-0.007	-0.003	-0.006
5	-0.007	-0.007	-0.004	-0.006	-0.01	-0.006	-0.006	-0.007
6	-0.006	-0.007	-0.004	-0.008	-0.007	-0.005	-0.004	-0.008
7	-0.008	-0.008	-0.004	-0.005	-0.007	-0.007	-0.007	-0.007
8	0.007	-0.006	-0.005	-0.004	-0.005	-0.005	-0.005	-0.005
9	-0.009	-0.007	-0.006	-0.006	-0.007	-0.007	-0.008	-0.009
10	-0.005	-0.007	-0.004	-0.005	-0.007	-0.007	-0.004	-0.007
11	-0.008	-0.005	-0.005	-0.007	-0.008	-0.008	-0.005	-0.008
12	-0.008	-0.004	-0.006	-0.007	-0.006	-0.006	-0.005	-0.008

Figure S12. Negative control monoclonal phage ELISA for pecan133 anti-BoNT A clones

Monoclonal phage ELISA signals of 96 clones of pecan133/134 immune heptaplex library first round selections on 1 μgmL^{-1} negative control antigen ovalbumin.

	A	B	C	D	E	F	G	H
1	-0.004	-0.008	-0.001	-0.004	-0.007	-0.008	-0.001	-0.004
2	-0.008	-0.009	0.001	-0.01	-0.012	-0.008	-0.008	-0.007
3	-0.009	-0.009	-0.004	-0.007	-0.007	-0.008	-0.003	-0.005
4	-0.009	-0.009	-0.002	-0.006	-0.009	-0.007	-0.006	-0.007
5	-0.011	-0.009	-0.003	-0.006	-0.007	-0.007	-0.008	-0.007
6	-0.008	-0.007	-0.003	-0.005	-0.007	-0.007	-0.004	-0.007
7	-0.009	-0.009	-0.001	-0.005	-0.007	-0.008	-0.006	-0.005
8	0.03	0.002	0.001	0.001	-0.007	-0.002	-0.005	-0.006
9	0.033	0.013	-0.002	-0.006	0.03	0.007	0	-0.006
10	0	-0.001	-0.002	0.005	-0.005	-0.005	-0.001	0.001
11	-0.007	-0.006	-0.003	-0.004	-0.004	-0.006	-0.005	-0.003
12	-0.004	-0.001	0.029	-0.005	-0.004	-0.006	-0.003	-0.007

Figure S13. Rapid antibody pairing of pecan126 selected anti-BoNT sdAb assayed on control solution phase protein

Negative control antigen ovalbumin 1 µg/mL used alongside the experimental pairing reveals no background signals after pairing sdAb clones isolated from the pecan 126 mediated retrofit of the heptaplex BoNT immune library.

	A1	A2	A3	B2	C6	D3	F1	G1	G3	H2	H8	H9	< TRACER
A1	0.01	0.01	0.006	0.004	0.001	-0.003	-0.002	-0.004	-0.011	-0.011	-0.011	-0.01	
A2	0.007	0	0.003	0.001	-0.004	-0.006	-0.006	-0.007	-0.012	-0.014	-0.013	-0.016	
A3	0.016	0.013	0.011	0.009	0.008	0.005	0.002	-0.005	-0.005	-0.006	-0.007	-0.011	
B2	0.017	0.015	0.019	0.004	0.004	0.005	0.003	0.002	-0.001	0	0.001	-0.002	
C6	0.001	0.004	0	0.003	-0.001	-0.001	0.001	-0.002	-0.002	-0.002	-0.001	-0.005	
D3	0	0	-0.002	0.001	-0.002	-0.002	-0.002	-0.004	-0.003	-0.004	-0.003	-0.006	
F1	0.003	0.005	0.004	0.003	0.005	0.002	0.006	0.002	0.001	0.002	0.005	0.011	
G1	-0.004	0.002	0.001	0.002	0.002	0.001	0.002	0.001	0.002	0.002	0.001	-0.002	
G3	0.003	-0.002	-0.002	0.002	-0.001	-0.001	0	-0.003	-0.006	-0.004	-0.006	-0.003	
H2	0.002	-0.002	-0.001	-0.002	-0.004	-0.003	-0.001	-0.002	-0.004	-0.002	-0.001	0.002	
H8	0.011	0.009	0.003	0.001	0.003	0.001	-0.001	0	0	0	0.001	0.001	
H9	0	0.001	-0.002	-0.004	-0.001	0	-0.001	-0.002	0	-0.003	-0.001	0	
CAPTOR													

Figure S14. Rapid antibody pairing of pecan133 selected anti-BoNT sdAb assayed on control solution phase protein

Negative control antigen ovalbumin 1 µg/mL used alongside the experimental pairing reveals no background signals after pairing sdAb clones isolated from the pecan133/134 mediated retrofit of a heptaplex BoNT immune library.

	A1	A4	A5	A6	B1	B3	B8	B9	B10	C11	E8	F1	<TRACER
A1	0.009	0.014	0.008	0.01	0.012	0.007	0.007	0.007	0.009	0.015	0.011	0.015	
A4	0.004	0.009	0.003	0.012	0.006	0.005	0.005	0.001	0.004	0.014	0.005	0.007	
A5	0.011	0.015	0.012	0.021	0.014	0.008	0.01	0.013	0.014	0.022	0.009	0.012	
A6	0.024	0.027	0.02	0.03	0.023	0.024	0.019	0.022	0.019	0.023	0.018	0.019	
B1	0.01	0.006	0.003	0.004	0.004	-0.001	-0.001	0	-0.001	0.005	0.002	0.003	
B3	0.009	0.009	0.004	0.004	-0.001	-0.003	-0.004	-0.003	-0.002	0.007	0	0.005	
B8	0.016	0.013	0.009	0.012	0.008	0.006	0.003	0.004	0.005	0.011	0.004	0.009	
B9	0.011	0.015	0.008	0.014	0.008	0.005	0.004	0.005	0.005	0.01	0.008	0.007	
B10	0.013	0.015	0.015	0.019	0.015	0.008	0.01	0.012	0.011	0.015	0.007	0.009	
C11	0.006	0.005	0.004	0.01	0.008	0.004	0.007	0.004	0.003	0.012	0.004	0.003	
E8	0.004	0.009	0.004	0.012	0.012	0.015	0.009	0.009	0.006	0.016	0.007	0.007	
F1	0.006	0.005	0.004	0.009	0.006	0.005	0.004	0.003	0.001	0.006	0.004	0.002	
CAPTOR													

Figure S15. Monoclonal phage ELISA of anti-*Ebolavirus* Zaire clones on negative control antigen

48 clones from round 3 and round 4 were screened on negative control antigen (1e+4 pfu per well) Marburg Musoke alongside the experimental screen.

	1	2	3	4	5	6	7	8	9	10	11	12
A	3.4E+04	2.8E+04	2.5E+04	2.5E+04	2.4E+04	2.4E+04	2.5E+04	2.5E+04	2.3E+04	8.6E+04	3.9E+04	2.3E+04
B	2.2E+04	2.0E+04	1.9E+04	2.5E+04	2.0E+04	2.1E+04	1.9E+04	2.4E+04	3.2E+04	4.5E+04	1.9E+04	2.2E+04
C	2.9E+04	2.0E+04	2.3E+04	9.2E+04	2.1E+04	1.8E+04	1.9E+04	2.0E+04	1.7E+04	2.3E+04	1.2E+05	2.3E+04
D	3.6E+04	3.0E+04	1.8E+04	3.9E+04	2.3E+04	2.7E+04	2.6E+04	2.9E+04	2.2E+04	2.4E+04	5.2E+04	2.5E+04
E	3.8E+04	2.5E+04	2.2E+04	2.5E+04	6.4E+04	3.6E+04	3.0E+04	3.2E+04	3.2E+04	6.7E+04	2.6E+04	2.7E+04
F	3.5E+04	2.5E+04	3.7E+04	3.5E+04	1.8E+05	3.8E+04	3.5E+04	3.2E+04	4.3E+04	2.5E+04	1.9E+04	3.4E+04
G	3.4E+04	2.8E+04	3.1E+04	8.5E+04	3.2E+04	3.5E+04	3.1E+04	3.6E+04	3.1E+04	3.4E+04	3.3E+04	3.6E+04
H	5.0E+04	2.9E+04	2.3E+04	2.9E+04	3.5E+04	4.2E+04	6.3E+04	3.9E+04	6.0E+04	2.6E+04	2.8E+04	4.6E+04
	Round 3						Round 4					

Supplementary methods

Construction of expression and display vectors

Non-biotinylating control vectors: pecan45[1] was modified to pecan73 by exchange of the lac for tac promoter using SOE-PCR, and insertion of PCR amplified MBG B sdAb from pecan21LgMBGB[2] to delete the hinge and fuse FR4 TVSS directly to the *Not*I site AAA reading frame to provide a basal hingeless pel-sdAb-His6 expression cassette. The full length g3p gene was PCR amplified from amberless pecan 21[3] using primer NotHisamberTEV (5'-gaattc,gcg,gcc,gca,cat,cac,cat,cac,cat,cac,tag,ggt,ggc,ggt,gga,tct,gag,aat,ctt,tat,ttt,cag,ggc,gga,ggt,ggc,ggt,gct,gaa,act,gtt,gaa,agt,tgt-3') encoding His6 tag, amber codon plus a TEV protease cleavage site, and back primer g3pTAAHind3 (3'-tat,gac,gca,tta,ttc,ctc,aga,att.att.cgaaaaaa-5'), and inserted into the *Not*I and *Hind*III sites of pecan73 to provide the basal phage display vector pecan114. The g3p gene was sequenced using primers lacZ' (5'-ctatgaccatgattacgaatttctag-3'), AHX228 (5'-cttatatcaaccctctcgacggc-3'), AHX229 (5'-tcgtttgtgaatatcaaggcc-3') and AHX230 (5'-cattggtgacgtttccggcc-3').

Biotin acceptor peptide insertions: Pecan122 was made by replacing g3p from pecan114 with a re-amplified product using front primer NotBAPHisg3p (5'-ca,gcg,gcc,gca,gga,ggc,ggt,gga,tct,ggc,ctg,aac,gat,att,ttc,gaa,gct,cag,aaa,atc,gaa,tgg,cac,gag,ggc,ggt,gga,ggc,tct,cat,cac,cat,cac,cat,cac,tag-3') and back primer g3pTAAHind3 to incorporate the minimal BAP[4] (underlined) flanked by Gly4Ser coding sequences. BAP linker variants employed a similar strategy but switching out the front primers with ones lacking the front (NotBAPG4SHisg3p), back (NotG4SBAPHisg3p) or both (NotBAPHisg3p Nolink) Gly4Ser coding sequences. G3p and linker sequences were verified as before.

Biotin ligase gene insertions: The *birA* second cistron was PCR amplified from a single µL of an overnight culture of *E. coli* DH10B[5], inserted into the *HindIII* site of pecan122 and orientation mapped wrt internal *Pst*I and the FR1 *Pst*I of the sdAb. The front primer for full-length *birA* was H3birAFOR (5'-aaaagcttaggaggacagctatg,aag,gat,aac,acc,gtg,cca-3') and for the deletion mutant[6] was H3MaedaFOR (5'-aaagcttaggaggacagctatg,atc,cag,tta,ctt,aat,gct,aaa,c-3') initiating to Ile64 with the T7 gene 10 ribosome binding sites underlined. For the low expression versions the ATG initiation codon was changed to GTG to encode H3birAgtgFOR and H3MaedagtgFOR respectively. The common back primer birAH3BACK (3'-g,gac,gca,tca,cgt,ctt,ttt,att.ttcgaaaa-5') was used for all *birA* formats. The various *birA* cistrons were sequenced with AHX89 (5'- cgcagtagcggtaaacggc-3') and AHX230.

Direct supE expression vectors: The starting *supE* tRNA sequence was initially synthesized as two complementary oligonucleotides supE top (5'-gtggggtatcgccaagcggtaaggcaccggattctaattccggcattccgaggttcgaatcctcgtaccccagccaa-3') and supE bottom (5'-agctttggctggggtacgaggattcgaacctcggaatgccggaattagaatccggtgccttaccgcttggcgataccccactgca-3') based upon Genbank M10708[7]. These were phosphorylated, annealed and bridged into *Pst*I / *Hind*III digested pAR3[8] (a generous gift of Dr. Julio Gutiérrez) to make pAR3supE. Low copy number of the vector dictated PCR amplification of the inserts by flanking primers and subsequent sequencing of the PCR product. pSCUPER was assembled by ligating native Pfu polymerase (Stratagene) amplified segments of

both pSC101 and pAR3supE in PEG ligation buffer in the presence of T4 PNK. Segments were generated as follows: pSC101 (ATCC, Manasas, VA) was first amplified using PacPSC101BACK (5’-aaattaattaagacagtaagacgggtaagcc-3’) with E93Rbottom (3’-ccaattccgaaagcctaaaggtcacctg-5’), and PacPSC101FOR (5’-cccaattaattatgatttttccccacgggag-3’) with E93Rtop (5’-ggttaaaggctttcggattttccagtggac-3’). The products were then fused by SOE-PCR using the Pac primers for pull-through to create a 2.1 kbp high copy number pSC101 origin[9] segment. pAR3-supE was amplified using PacPAR3BACK (5’-aaattaattaacctgaagtcagccccatacg-3’) and PacPAR3FOR (5’-cccaattaattccgaataaatacctgtgacgg-3’) to create the 2.0 kbp arabinose inducible supE segment bearing the chloramphenicol resistance gene. Here, sequencing was replaced by phenotypic screening in two ways. First, clones were benchmarked against the original high copy number pSC101 mutants (generous gifts of Dr. Gregory Phillips) for approximate copy number equivalence by agarose gel electrophoresis. Second, clones were mobilized to DH10F’tet with pecan114 to screen for suppression in the presence of 2% glucose, 2000 $\mu g mL^{-1}$ arabinose and 1 mM IPTG following superinfection with M13K07 and kanamycin selection. Suppression to enable MBG B sdAb display was monitored by phage ELISA with positives scored for signal on *Marburgvirus* NP and not *Ebolavirus* NP, and the highest signal to noise clone (#9) taken forward. pSCUPERV88 and pSCUPERV89 were made by replacing the wild type supE region between the *Pst*I and *Hind*III sites of pSCUPER with phosphorylated and annealed bridges comprising either V88 top (5’-gtggggtatcgccaagcggtaaggcaccggattctaactccggcattccgaggttcgaatcctcgtaccccagccaa-3’) plus V88 bottom (5’-agctttggctggggtacgaggattcgaacctcggaatgccggagttagaatccggtgccttaccgcttggcgataccccactgca-3’) or V89 top (5’-gtggggtatcgccaagcggtaaggcaccggattctaaatccggcattccgaggttcgaatcctcgtaccccagccaa-3’) plus V89 bottom (5’-agctttggctggggtacgaggattcgaacctcggaatgccggatttagaatccggtgccttaccgcttggcgataccccactgca-3’).

Transdisplay platform vectors: g7p, g8p and g9p genes were amplified from M13K07 replicative form in a similar manner to the g3p gene using front primers NotHisAmberTEV-g7p, -g8p and -g9p which had the common 5’-region encoding *Not*I-His6-amber-TEV but varied in their priming sequences according to the gene targeted; g7p –atg,gag,cag,gtc,gcg,gat,ttc-3’; g8p –gct,gag,ggt,gac,gat,ccc-3’; g9p-atg,agt,gtt,tta,gtg,tat,tct,ttc. The back primers were g7pTAAHind3 (3’-t,tag,cga,ccc,cca,gtt,cct.att.att.cgaaaaa-5’), g8pTAAHind3 (3’-tgg,agc,ttt,cgt,tcg.att.att.cgaaaaaa) and g9pTAAHind3 (3’-gg,gca,aat,tac,ctt,tga,agg,agt.att.att.cgaaaaaa-5’). Products were used to replace g3p in pecan114 *via Not*I and *Hind*III and sequenced with primer lacZ’ to create pecan115 (MBGB sdAb-gp9), 119 (MBGB sdAb-gp7) and pecan120 (MBGBsdAb-g8p). A synthetic *E. coli* optimized core streptavidin gene from Glu13 to Ser139[10] with flanking *Nco*I (encoding AMA) and *Not*I (encoding AAA) sites and based upon Genbank X03591.1[11] was used to replace the sdAb in pecan114, 115, 119 and 120 to make the strep-display versions with clones sequenced using lacZ’ primer.

Strep-display cassettes were PCR amplified using PstV88lacZ (5’-taactgcagtggggtatcgccaagcggtaaggcaccggattctaactccggcattccgaggttcgaatcctcgtaccccagccaatttattcaagacgcttaccttgtaagtgcacccagtctatgaccatgattacgatct-3’) which encodes the V88 supE mutant and intervening region between the two Gln_2 tRNAs[12] and the common back sequencing primer AHX89 from respective strep-gp vectors and used to replace the resident supEv88 insert within pSCUPERV88 *via Pst*I and *Hind*III.

Transdisplay sdAb vectors: pecan133 was derived from pecan126 by replacing the resident g3p and GTGΔbirA cistron region *via Not*I and *Hind*III with a re-amplified GTGΔbirA region made with Not126Asc (5'-tca,gcg,gcc,gca,gga,ggc,ggt,gga,tct,ggc,ctg,aac,gat,att,ttc,gaa,gct,cag,aaa,atc,gaa,tgg,cac,gag,ggc,ggt,gga,ggc,tct,cat,ca,cat,cac,cat,cac,taa.taa.ggcgcgccttaggaggacagctgtg,atcc-3') and AHX89 which fuses the G4SBAPG4S-His6 region to the *Not*I site and two ochre termination codons and re-encodes the rbs and GTG initiation region for ΔbirA.

Hygromycin resistant sdAb transdisplay vector: The hygromycin gene was a generous gift of Dr. Jim Sweigard and was isolated from pCB1004 by ligation in PCR using HygFRONTSwa (5'-aaggaaatttaa.atg,aaa,aag,cct,gaa,ctc,ac-3'), HygBACKSwa (3'-ca,ggc,tcc,cgt,ttc,ctt,att.taaatttatccgt-5'), HygNcoDEL (5'-cag,ccg,gtc,gcg,gag,gcT,atg,gat,gcg,atc,gct,g-3') and HygNdeDEL (5'-ca,tgg,cgt,gat,ttc,atC,tgc,gcg,att,gct,ga,cc) to delete internal *Nco*I and *Nde*I restrictions sites and inserted in place of the ampicillin resistance gene in pecan 133 *via Swa*I to make pecan 164.

Antigens

Arrays of *Marburgvirus* NP offering a potentially polyvalent surrogate have been demonstrated previously when overexpressed in the *E. coli* cytosol [13] and we assembled an untagged Musoke NP gene and Ebola Zaire strain Kikwit 1995 gene[2], and overexpressed them using pecan42 in Tuner+pRARE. Immunoblotting and probing with sdAb-AP fusions were first used to verify small-scale expression which was then scaled up to 2x400 mL in glucose free terrific broth with induction for 6 h at 1 mM IPTG at 25 ^{o}C, yielding approx. 20 g wet weight total. Pellets were resuspended in total volume of 100 mL for bead-beating in 100 mM TrisHCl pH7.5, 1 mM EDTA, Roche complete protease inhibitors, 0.05% Tween-20 and 1 mM DTT. Lysates were clarified by repeated centrifugation and decanting (Allegra GPR, 5.75 krpm, 30 min 4 ^{o}C), and finally filtration through a 0.22 µm vacuum filter and stored in 2 mL aliquots at -80 ^{o}C until required. ELISA titration of passively immobilized sdAb as captor and sdAb-AP as tracer in Tris buffered saline was used to verify the antigens were a suitable crude surrogate for polyvalent viral NP (supplementary **figure S1**) with 10 µL per well in MPBS determined to be our standard.

To produce NP for direct capture ELISA we used a similar harvesting approach applied to the C-terminally His tagged proteins in pecan42 to enable IMAC purification of denatured monomer. Beadbeating was in 100 mM TrisHCl pH 7.5, 100 mM NaCl, 5% glycerol, 6M guanidine HCl, 0.05% Tween-20, 10 mM β-mercapoethanol and 20 mM imidazole with protease inhibitor cocktail. Clarified and filtered supernatant was applied with a superloop to a 1 mL nickel sepharose FF column and protein eluted via a 20 mM to 600 mM imidazole gradient. Peak fractions were silver stained and western blotted with anti-His HRP revealing expected 100 kDa band as the dominant species which were pooled and stored in 100 µL fractions at -20 ^{o}C. Prior to use after thawing, the denatured preparation was microfuged to remove most of the aggregates that formed, with ELISA plate coating employing 1µL per mL PBS.

Botulinum neurotoxins were purchased from Metabiologics (Madison, Wisconsin). 1 μgmL^{-1} in PBS was employed for panning, ELISA coats and pairing.

Ebolavirus Zaire strain Kikwit 1995[2] was amplified in 16 x 225 cm^3 flasks of Vero cells in DMEM/5% FBS/penicillin/streptomycin for 4 days. The 40 mL supernatants were collected, gently clarified by centrifugation (Allegra 6R, swing-out, 2.5 krpm, 5 min, 4 ^oC) and stored at -80^0. Fresh media was added to the flasks and the amplification left for 4 more days. Day 4 supernatants were thawed at room temperature, day 8 supernatants were gently clarified by centrifugation and all virus was precipitated by addition of 1/5th volume of PEG 8000/2.5M NaCl at 4 ^oC overnight. Precipitated virus was pelleted (Allegra 6R, 3.5 krpm, 15 min, 4 ^oC), resuspended in a total of 4 mL PBS and 4 x 1 mL centrifuged through 10 % (w/v) sucrose/PBS onto 65 % sucrose cushions (Beckman L70M , SW40, 20 krpm, 1 h, 4^{0}C). Virus was harvested, diluted to 6 mL and loaded a top six 20 to 65 % continuous gradients, and centrifuged at 20 krpm for 24 h at 4 ^oC. Bands were made visible with a torch from above, harvested, pooled and dialyzed against 3 x 300 volume changes of PBS before aliquoting and storage at -80 ^oC. Virus was titrated by plaque formation on duplicate 6 well plates of Vero cells with a 1 h infection, gentle wash and overlay with 2mL of EMEM/5% FBS/penicillin/streptomycin containing 0.6% Seaplaque GTG agarose. On day 10, 2 mL 4% formaldehyde was added to each well. The plates were incubated in a closed box at 37 ^oC overnight. The agarose plugs were removed and the monolayer was stained with crystal violet for plaque visualization and counting. Coats of virus for the first round of panning were 8 x 100 μL each containing approximately 4e+5 pfu. Coats for rounds 2 through 4 were 8 x 25 μL virus + 75 μL PBS each containing approximately 1e+5 pfu. For polyclonal phage screening, wells were coated with approximately 4e+3 pfu and for monoclonal phage screening wells were coated with approximately 1e+4 pfu. *Marburgvirus* strain Musoke 1980 purified previously[2] was used as control virus. Viruses were incubated in a final concentration of 0.1% Triton in MPBS for 10 min before applying to the ELISA plate to release the nucleocapsid.

Mass spectrometry of proteins: 5 to 10 μg of protein was diluted ~10 fold into 0.2% TFA (10 μl ptn into 100 μl TFA). The sample was bound to a C4 ZipTip (Millipore) by aspirating and dispensing into a second tube labeled "flow through" ~6 times, 20 μl each. The C4 ZipTip was then washed with 100 μl 0.2% TFA, and eluted into 50 μl 50% acetonitrile; 0.5% acetic acid. The eluate was infused into a ThermoFinnigan Quantum triple quadrupole mass spectrometer at 0.6 μl/min *via* a 250 μl SGE glass syringe using the mass spectrometer's integrated syringe pump. A New Objective IntegraFrit (IF360-75-50-N-5) was used inline as a pre-filter before the sample reached the New Objective Column Adapter (ADPC-IMS), which replaces the standard ESI probe of the ThermoFinnigan Ion Max source, and introduced into the mass spectrometer *via* a New Objective PicoFrit (PF360-75-10-N-5) emitter. The instrument was set in positive ion mode. Spray voltage was 2000V, and the heated capillary was set at 250°C. Data was obtained using the full scan mode of quadrupole 1. Repeated snapshot scans were acquired from 150-1500 m/z. Q1 peak width was set between 1.2 to 4.0 depending upon the sample MW, and data processing was set to average between 20 to 50 spectra depending upon the peak intensity. Data files were opened in ThermoFinnigan's QualBrowser (v 2.0) and processed by using 'boxcar' type smoothing set at 7 points. The exact mass spectra was then exported to the clipboard and subsequently opened in

ProMass for Xcalibur (v 2.5 SR-1) for deconvolution. Baseline removal was set at low/normal, and smoothing was turned off. Comprehensive deconvolution settings were as follows: peak width 3; merge width 0.3; minimum score 0.2; normalize score 1. Result files were subsequently captured as screenshots and dropped into a PowerPoint presentation.

1. Conway, J.O., Sherwood, L.J., Collazo, M.T., Garza, J.A. & Hayhurst, A. Llama single domain antibodies specific for the 7 botulinum neurotoxin serotypes as heptaplex immunoreagents. *PLoS One* **5**, e8818 (2010).
2. Sherwood, L.J., Osborn, L.E., Carrion, R., Jr., Patterson, J.L. & Hayhurst, A. Rapid assembly of sensitive antigen-capture assays for Marburg virus, using in vitro selection of llama single-domain antibodies, at biosafety level 4. *J Infect Dis* **196 Suppl 2**, S213-219 (2007).
3. Goldman, E.R. et al. Facile generation of heat-stable antiviral and antitoxin single domain antibodies from a semisynthetic llama library. *Anal Chem* **78**, 8245-8255 (2006).
4. Beckett, D., Kovaleva, E. & Schatz, P.J. A minimal peptide substrate in biotin holoenzyme synthetase-catalyzed biotinylation. *Protein Sci* **8**, 921-929. (1999).
5. Durfee, T. et al. The complete genome sequence of Escherichia coli DH10B: insights into the biology of a laboratory workhorse. *J Bacteriol* **190**, 2597-2606 (2008).
6. Maeda, Y., Yoshino, T. & Matsunaga, T. In vivo biotinylation of bacterial magnetic particles by a truncated form of Escherichia coli biotin ligase and biotin acceptor peptide. *Applied and environmental microbiology* **76**, 5785-5790 (2010).
7. Inokuchi, H., Kodaira, M., Yamao, F. & Ozeki, H. Identification of transfer RNA suppressors in Escherichia coli. II. Duplicate genes for tRNA2Gln. *J Mol Biol* **132**, 663-677 (1979).
8. Perez-Perez, J. & Gutierrez, J. An arabinose-inducible expression vector, pAR3, compatible with ColE1-derived plasmids. *Gene* **158**, 141-142 (1995).
9. Peterson, J. & Phillips, G.J. New pSC101-derivative cloning vectors with elevated copy numbers. *Plasmid* **59**, 193-201 (2008).
10. Schmidt, T.G. & Skerra, A. One-step affinity purification of bacterially produced proteins by means of the "Strep tag" and immobilized recombinant core streptavidin. *J Chromatogr A* **676**, 337-345 (1994).
11. Argarana, C.E., Kuntz, I.D., Birken, S., Axel, R. & Cantor, C.R. Molecular cloning and nucleotide sequence of the streptavidin gene. *Nucleic Acids Res* **14**, 1871-1882 (1986).
12. Nakajima, N., Ozeki, H. & Shimura, Y. Organization and structure of an E. coli tRNA operon containing seven tRNA genes. *Cell* **23**, 239-249 (1981).
13. Kachko, A.V. et al. [Comparative study of the morphology and antigenic properties of recombinant analogs of a Marburg virus nucleoprotein]. *Mol Biol (Mosk)* **35**, 492-499 (2001).
